# A High-Fat Diet Increases Kidney Fibrosis Through Regulating TGF-β and PDGF-β Signaling Pathways in Normotensive and Hypertensive Rat Models

**DOI:** 10.3390/ijms26168031

**Published:** 2025-08-20

**Authors:** Fatema Binte Abdullah, Abdullah Md. Sheikh, Shatera Tabassum, Atsushi Nagai, Jun Yoshino, Takeshi Kanda, Toru Nabika, Shozo Yano

**Affiliations:** 1 Department of Nephrology, Faculty of Medicine, Shimane University, 89-1 Enya Cho, Izumo 693-8501, Japan; m239401@med.shimane-u.ac.jp (F.B.A.); jyoshino@med.shimane-u.ac.jp (J.Y.); t-kanda@med.shimane-u.ac.jp (T.K.); 2 Department of Laboratory Medicine, Faculty of Medicine, Shimane University, 89-1 Enya Cho, Izumo 693-8501, Japan; tabassum@med.shimane-u.ac.jp (S.T.); syano@med.shimane-u.ac.jp (S.Y.); 3 Department of Neurology, Faculty of Medicine, Shimane University, 89-1 Enya Cho, Izumo 693-8501, Japan; anagai@med.shimane-u.ac.jp; 4 School of Nursing, Faculty of Medicine, Shimane University, 89-1 Enya Cho, Izumo 693-8501, Japan; nabika@med.shimane-u.ac.jp

**Keywords:** hypertension, high-fat diet, kidney fibrosis, PDGF-β, TGF-β

## Abstract

Hypertension and obesity are well-established risk factors for chronic kidney disease (CKD). This study investigates the interaction between these two factors in CKD using animal models. Twelve-week-old normotensive Wistar Kyoto (WKY), spontaneously hypertensive (SHR), and stroke-prone spontaneously hypertensive (SHR-SP) rats were fed either a normal diet (control) or a high-fat diet (HFD) for eight weeks. Kidney pathology and molecular mechanisms were assessed via immunostaining, real-time PCR, and Western blotting. In the control-fed groups, SHR-SP showed the most severe glomerular and tubular fibrosis, followed by SHR. The HFD exacerbated fibrosis in both the WKY and SHR rats but not in the SHR-SP rats. The levels of the mesangial marker smooth muscle α-actin (SMA) in the glomeruli were highest in the control-fed SHR-SP rats. HFD feeding increased glomerular SMA levels in WKY and SHR but not in SHR-SP. The levels of the mesenchymal marker vimentin were elevated in the control-fed SHR-SP rats compared to the other control-fed animals. The HFD increased the vimentin levels in WKY but decreased them in SHR-SP. The HFD increased senescence and inflammatory markers in the kidneys of the WKY and SHR rats. The HFD-fed WKY and SHR rats also showed upregulation of platelet-derived growth factor β (PDGFβ) signaling molecules. Among the control-fed animals, the transforming growth factor β (TGFβ) and TGFβ receptor 2 (TGFβR2) levels were elevated in SHR-SP. HFD feeding increased the TGFβR2 levels in WKY and the SHR and TGFβ levels in WKY. Similarly, SMAD2/3 activation was the highest in the SHR-SP control group. HFD feeding increased the SMAD2/3 activation in the kidneys of the WKY and SHR rats. Thus, our findings demonstrate that a high-fat diet can intensify renal fibrosis independent of hypertension through TGFβ and PDGFβ signaling within a two-month timeframe.

## 1. Introduction

Chronic kidney disease (CKD) refers to a group of disorders characterized by the gradual and progressive loss of kidney function due to structural and functional alterations in the organ [1]. This condition affects approximately 10% of the global population, and its prevalence is steadily increasing [2]. It is classified into five stages based on kidney function, as determined by the glomerular filtration rate (GFR) and albuminuria levels [3]. The CKD umbrella includes a variety of renal pathologies, such as glomerulonephritis, polycystic kidney disease, chronic interstitial nephritis, and focal segmental glomerulosclerosis (FSGS) [4,5,6,7,8]. CKD can arise as a consequence of systemic conditions like hypertension, diabetes mellitus, autoimmune disorders, cardiovascular disease, and metabolic syndromes [9,10,11,12,13]. Despite this wide range of etiologies, fibrosis is a unifying pathological hallmark of CKD and is considered one of the principal mechanisms driving progressive renal dysfunction [14].

Hypertension is one of the most common and significant risk factors for CKD [9]. It can directly contribute to renal injury by promoting both glomerular and tubulointerstitial fibrosis. Although the precise mechanisms through which hypertension induces kidney fibrosis are not fully understood, accumulating evidence suggests a strong association with the activation of the renin–angiotensin–aldosterone system (RAAS), a key regulator of blood pressure and fluid balance [15,16]. Under hypertensive conditions, dysregulation of the RAAS contributes to fibrotic progression by promoting vasoconstriction, stimulating the production of proinflammatory cytokines, and enhancing extracellular matrix accumulation [15]. In addition, hypertension is believed to cause glomerular vascular barotrauma, which initiates chronic inflammation and fibrotic remodeling [17]. This barotrauma can also extend to the vasculature within the tubulointerstitial compartment, further exacerbating renal injury. One protective response against such hypertension-induced damage is renal autoregulation, in which the afferent arterioles constrict in response to elevated blood pressure [18]. This vasoconstriction reduces the mechanical stress on the glomerular structures, helping to limit injury. However, persistent or severe hypertension can overwhelm this compensatory mechanism [17,18]. Indeed, studies have shown that uncomplicated or moderate hypertension typically results in minimal or “benign” fibrosis unless there is a sustained and significant elevation in blood pressure [19]. However, the presence of comorbid conditions such as diabetes or metabolic syndrome in hypertensive individuals substantially amplifies the risk of kidney fibrosis [20,21]. In such cases, even minor fluctuations in blood pressure can provoke disproportionately severe fibrotic changes.

Hypertension frequently coexists with obesity, a condition often associated with Western dietary patterns rich in saturated fats and refined carbohydrates [22]. This diet-induced obesity promotes a state of low-grade systemic inflammation and insulin resistance, both of which elevate vascular risk by damaging the endothelium [23,24]. Diets high in saturated fats also contribute to dyslipidemia, which disrupts vascular homeostasis by increasing the production of reactive oxygen species (ROS) and proinflammatory mediators such as tumor necrosis factor-alpha (TNF-α), interleukin-6 (IL-6), and adhesion molecules like vascular cell adhesion molecule-1 (VCAM-1) and intercellular adhesion molecule-1 (ICAM-1) [25,26]. Being highly vascular organs, the kidneys are particularly susceptible to such insults. Dyslipidemia further contributes to renal injury through lipotoxicity, the pathological accumulation of fatty acids and triglycerides in non-adipose tissues, particularly in renal tubular epithelial cells and podocytes [27,28]. CKD phenotypes associated with lipotoxicity include diabetic nephropathy, obesity-related glomerulopathy, and age-related kidney dysfunction. Lipotoxicity induces mitochondrial dysfunction, oxidative stress, cellular senescence, and inflammation, all of which contribute to fibrotic remodeling in the kidneys [27,28]. In addition, obesity impairs kidney function through the release of nephrotoxic adipokines and cytokines from the adipose tissue, promoting local inflammation and fibrosis [29]. Hence, hypertension and a high saturated fat intake share overlapping pathogenic mechanisms, including vascular injury, oxidative stress, and proinflammatory signaling, that can converge to drive kidney fibrosis.

This study aimed to investigate the early-stage interaction between high saturated fat intake and hypertension in promoting renal fibrosis. Notably, while hypertension alone typically leads to benign fibrosis, coexisting obesity may significantly accelerate disease progression through inflammatory, hemodynamic, and metabolic alterations, including activation of the RAAS pathway. To explore this interplay, we examined three rat strains: normotensive Wistar Kyoto (WKY) rats, stroke-resistant spontaneously hypertensive rats (SHR), and stroke-prone spontaneously hypertensive rats (SHR-SP). SHR-SP rats are genetically predisposed to severe hypertension and kidney fibrosis, whereas SHR rats develop moderate hypertension with minimal fibrotic damage. All rats were fed a high-saturated-fat diet for eight weeks. Our results demonstrated that the high-saturated-fat diet induced kidney fibrosis in both WKY and SHR rats to a similar extent, whereas the SHR-SP rats did not show further increases in fibrosis, likely due to an already maximal fibrotic state. These findings suggest that dietary saturated fats can initiate or exacerbate kidney fibrosis through mechanisms that may be at least partially independent of hypertension in the early stages. They also emphasize the additive or synergistic effects of dietary and hemodynamic stressors in CKD pathogenesis.

## 2. Results

### 2.1. The Effects of a High-Fat Diet on Kidney Histology and Fibrosis in Normotensive and Hypertensive Rat Models

To examine the effects of a high-fat diet on the kidneys, normotensive Wistar Kyoto (WKY) rats and hypertensive and spontaneously hypertensive rats (SHR and SHR-SP) were fed either a normal rat chow diet (control diet) or a high-fat diet (HFD), where 60% of the calories came from lard. Body weight increased gradually in a time-dependent manner in both diet groups; however, the rate of increase was greater in the high-fat diet groups [30]. When comparing the WKY, SHR, and SHR-SP rats, the rate of body weight gain was lower in the SHR-SP rats than that in the other two strains, in both the control and high-fat-diet groups [30]

**Figure 1:**
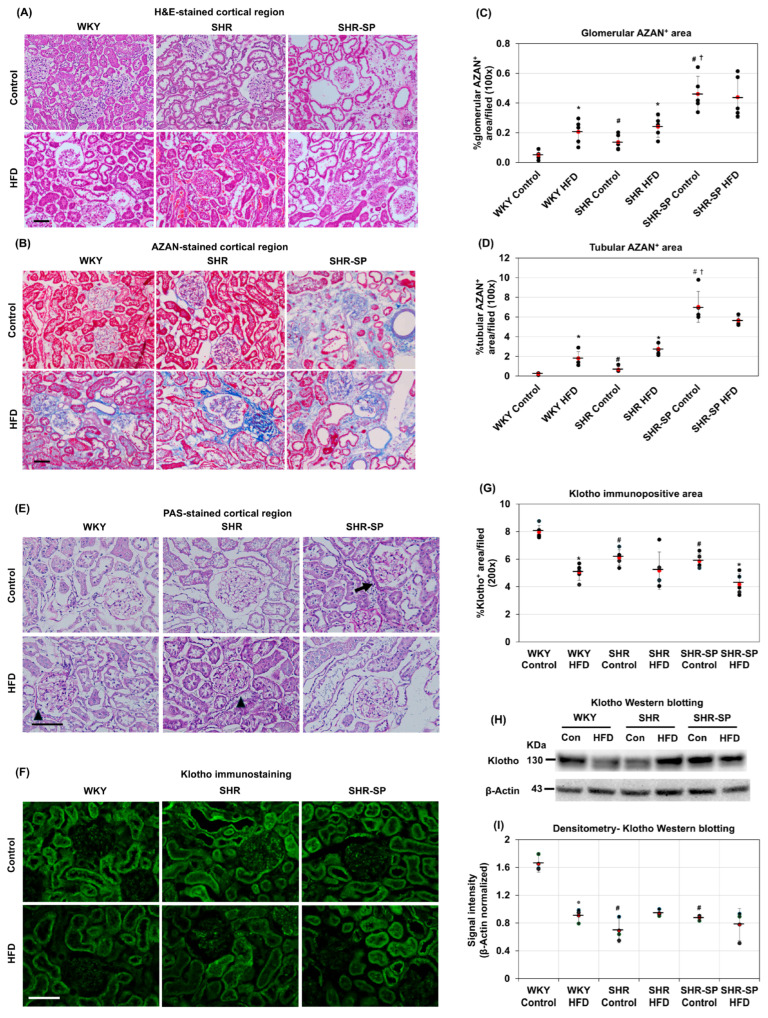
The effects of a high-fat diet on kidney histology and fibrosis. To examine the effects of a high-fat diet on kidney structure, hematoxylin and eosin (H&E) staining was performed. Representative photomicrographs of H&E-stained cortical regions of WKY, SHR, and SHR-SP rats fed either a control diet or a high-fat diet are shown in panel (**A**). Fibrotic changes were assessed using AZAN staining. Panel (**B**) shows representative photomicrographs of AZAN-stained cortical regions of the kidney. The extent of fibrosis in the glomerulus and tubular interstitial spaces was quantified. The average fibrotic areas, expressed in %area from photomicrographs taken at 100× magnification, in the glomerulus and tubular interstitial regions are displayed in panels (**C**) and (**D**), respectively (*n* = 5). To check the effects of a high-fat diet on kidney injury, PAS staining and Klotho immunostaining were performed. Representative photomicrographs of PAS-stained cortical regions of the kidneys are shown in (**E**). (**F**) Klotho immunostaining photomicrographs are shown here. The extent of the immunopositive areas was quantified. The average Klotho immunopositive areas, expressed in %area from photomicrographs taken at 200× magnification, are displayed in panels (**G**) (*n* = 5). The levels of Klotho were assessed further using Western blotting. A representative blot in Klotho Western blotting is shown in (**H**). β-actin was used as a loading control. The intensities of the Klotho Western blotting bands were quantified through densitometry, and the averages (*n* = 3) of the β-actin normalized intensities are shown in (**I**). The numerical data are presented as dot-plots with averages (marked by red dots with a line) and ± SD. Statistical significance is denoted as follows: * *p* < 0.05 compared to control-diet-fed rats of the same strain; ^#^ *p* < 0.05 compared to control-diet-fed WKY rats; ^†^ *p* < 0.05 compared to control-diet-fed SHR rats. Bar = 100 µm.

To assess kidney histology, HE staining was performed. In the WKY rats fed a control diet, the glomeruli and tubules were well formed (Figure 1A). However, in the WKY and SHR rats fed an HFD, the tubular cells appeared swollen. Some red blood cells (RBCs) were detected in both the glomeruli and the tubular interstitial spaces in the SHR rats on a control diet but not in WKY, while they were increased in SHR-SP. Considerable numbers of RBCs were detected in both the WKY and SHR rats fed the HFD, and RBCs were more frequently observed in the HFD-fed SHR rats than in the WKY rats. In the SHR-SP rats fed an HFD, the RBC accumulation did not increase compared to that in the control-diet-fed animals (Figure 1A). At high magnification (×1000), necrotic changes were evident in the tubular epithelial cells of the HFD-fed animals (Supplemental Figure S1). Additionally, fibrotic changes were frequently observed in the SHR-SP rats, regardless of diet.

To analyze kidney fibrosis, AZAN staining was performed. The results showed that AZAN-positive areas (blue) were barely detectable in the cortex of the kidneys from the WKY rats fed a control diet (Figure 1B). A slight but significantly higher presence of AZAN-positive areas in the glomerulus and tubular interstitial spaces was observed in the control-diet-fed SHR rats. In SHR-SP, extensive AZAN-positive fibrotic areas were observed in the cortex of the control-diet-fed rats, which were greater than those in the control-diet- or HFD-fed WKY and SHR rats (glomerulus (%/field): WKY control: 0.05 ± 0.03; SHR control: 0.14 ± 0.05; SHR-SP: 0.46 ± 0.12 (*p* values: WKY control vs. SHR control: <0.012; WKY control vs. SHR-SP control: <0.0001; SHR control vs. SHR-SP control: <0.001); interstitial: WKY control: 0.22 ± 07; SHR control: 0.67 ± 0.28; SHR-SP control: 7.04 ± 1.58 (*p* values: WKY control vs. SHR control: <0.001; WKY control vs. SHR-SP control: <0.0001; SHR control vs. SHR-SP control: <0.0001)) (Figure 1B–D). When the rats were fed an HFD, the AZAN-positive areas in the cortex were increased in both the WKY and SHR rats (glomerulus (%/field): WKY control: 0.05 ± 0.03; WKY HFD: 0.21 ± 0.08; SHR control: 0.14 ± 0.05; SHR HFD: 0.24 ± 0.07; SHR-SP control: 0.46 ± 0.12; SHR-SP HFD: 0.44 ± 0.14 (*p* values: WKY control vs. WKY HFD: <0.004; SHR control vs. SHR HFD: <0.03; SHR-SP control vs. SHR-SP HFD: 0.79); interstitial: WKY control: 0.22 ± 007; WKY HFD: 1.8 ± 0.67; SHR control: 0.67 ± 0.28; SHR HFD: 2.78 ± 0.59; SHR-SP control: 7.04 ± 1.58; SHR-SP HFD: 5.63 ± 0.42 (*p* values: WKY control vs. WKY HFD: <0.001; SHR control vs. SHR HFD: <0.0001; SHR-SP control vs. SHR-SP HFD: 0.09)) (Figure 1C,D). However, in SHR-SP, AZAN-positive areas did not increase but rather decreased with an HFD, although this difference did not reach a significant level. Nonetheless, their levels remained higher compared to those in WKY and SHR, regardless of diet (Figure 1C,D).

### 2.2. The Effects of a High-Fat Diet on Kidney Injury in Normotensive and Hypertensive Rat Models

To analyze kidney injury, PAS staining was performed. The results showed that the Bowman’s capsules and tubular basement membranes of the WKY rats fed a control diet were thin, with well-defined urinary space areas. In the kidneys of the SHR rats fed a control diet, the Bowman’s capsules and tubular basement membranes were slightly thicker than those in the WKY rats and were markedly thick in the SHR-SP rats fed a control diet (Figure 1E, arow). HFD feeding increased the PAS-positive areas in the Bowman’s capsules and tubular basement regions in both the WKY and SHR rats but not in the SHR-SP rats (Figure 1E, arrowhead).

Klotho plays a crucial role in maintaining mineral homeostasis, providing antioxidative defense, and inhibiting fibrosis and inflammation [31]. Therefore, a reduction in Klotho is strongly associated with kidney dysfunction and may contribute to kidney damage. To investigate the effects of the HFD on Klotho levels, we examined its expression in the renal cortex of the WKY, SHR, and SHR-SP rats. The immunostaining results showed that in the WKY rats fed a control diet, nearly all of the renal tubules were positive for Klotho (Figure 1F). In contrast, not all of the tubules in the SHR and SHR-SP rats exhibited Klotho positivity (Figure 1F,G). Importantly, all strains fed with an HFD showed a reduction in Klotho-positive areas compared to those in their control-diet-fed counterparts, although this reduction was not statistically significant in the SHR rats ((%/field): WKY control: 7.97 ± 0.5; WKY HFD: 5.05 ± 0.58; SHR control: 6.11 ± 0.57; SHR HFD: 5.16 ± 1.35; SHR-SP control: 5.86 ± 0.53; SHR-SP HFD: 4.18 ± 0.76 (*p* values: WKY control vs. WKY HFD: 0.00005; SHR control vs. SHR HFD: 0.186; SHR-SP control vs. SHR-SP HFD: <0.004; WKY control vs. SHR control: <0.001; WKY control vs. SHR-SP control: <0.0005)) (Figure 1G).

To analyze the Klotho levels further, Western blotting was performed. The results revealed that the Klotho levels in the cortex were highest in the control-diet-fed WKY rats compared to those in the SHR and SHR-SP rats. When comparing the HFD-fed animals, a significant reduction in Klotho levels was observed only in the WKY rats, whereas the SHR and SHR-SP rats showed no significant difference compared to their control-diet-fed counterparts (Figure 1H,I).

### 2.3. The Effects of a High-Fat Diet on Kidney Fibrosis Types in Normotensive and Hypertensive Rat Models

**Figure 2:**
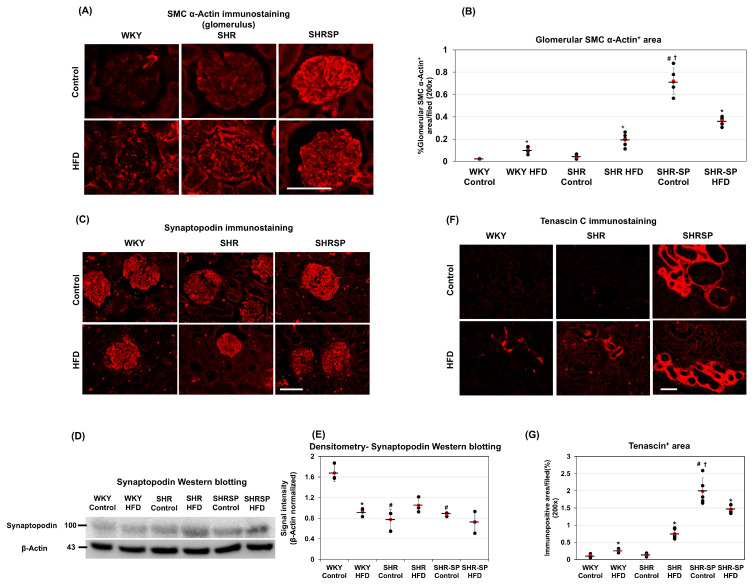
The effects of a high-fat diet on types of kidney fibrosis. To evaluate the impact of a high-fat diet on kidney fibrosis in WKY, SHR, and SHR-SP rats, markers for mesenchymal cells (SMC α-actin), podocytes (synaptopodin), and fibrosis-related proteins (tenascin c) were assessed. Immunostaining photomicrographs depicting SMC-α-actin-positive cells within the glomeruli are shown in (**A**). SMC-α-actin-positive areas in the glomeruli were quantified, expressed in %area from photomicrographs captured at 200× magnification, and the average immunopositive areas are shown in (**B**) (*n* = 5). (**C**) Representative immunostaining photomicrographs illustrating synaptopodin are shown here. Synaptopodin levels were analyzed further through Western blotting, with representative blots provided in (**D**). In (**E**), the average signal intensities of the synaptopodin blots are presented. β-actin served as a loading control (*n* = 3). (**F**) Representative photomicrographs of tenascin-c-immunostained cortical areas in the kidneys are shown here. Tenascin-c-positive areas were quantified using ImageJ software, expressed in %area from photomicrographs at 200× magnification, and the average immunopositive areas are shown in (**G**) (*n* = 5). The numerical data are presented as dot-plots with averages (marked by red dots with a line) and the ± SD. Statistical significance is indicated as follows: * *p* < 0.05 compared to control-diet-fed rats of the same strain; ^#^ *p* < 0.05 compared to control-diet-fed WKY rats; ^†^ *p* < 0.05 compared to control-diet-fed SHR rats. Bar = 100 µm.

The proliferation of mesenchymal cells, including myofibroblasts and mesangial cells, is closely associated with glomerulosclerosis [32]. Additionally, α-SMA is a well-established marker of activated mesenchymal cells involved in fibrosis, including glomerular fibrosis [32]. Therefore, we investigated the α-SMA expression levels in the glomeruli in different rat strains. The immunostaining results showed that α-SMA was faintly positive in the glomeruli of the WKY rats fed a control diet (Figure 2A). In the SHR rats fed a control diet, the α-SMA-positive areas in the glomeruli were slightly but significantly greater compared to those in the control-diet-fed WKY rats ((%/field): WKY control: 0.023 ± 0.002; WKY HFD: 0.103 ± 0.029; SHR control: 0.04 ± 0.018; SHR HFD: 0.193 ± 0.06; SHR-SP control: 0.721 ± 0.117; SHR-SP HFD: 0.363 ± 0.0.041 (*p* values: WKY control vs. WKY HFD: <0.005; SHR control vs. SHR HFD: <0.001; SHR-SP control vs. SHR-SP HFD: <0.0005; WKY control vs. SHR control: 0.07; WKY control vs. SHR-SP control: <0.000005; SHR control vs. SHR-SP control: <0.000005)) (Figure 2B and Supplemental Figure S2). In both the WKY and SHR rats, the α-SMA-positive areas were increased in response to the HFD compared to those in their control-diet-fed counterparts (Figure 2B and Supplemental Figure S2). However, there was no significant difference in α-SMA expression between the HFD-fed WKY and HFD-fed SHR rats. In the SHR-SP rats, the α-SMA levels were markedly higher than those in WKY and SHR, regardless of diet. Interestingly, the HFD treatment did not increase the α-SMA levels in the SHR-SP rats; instead, its expression decreased compared to that in the control-diet-fed SHR-SP rats (Figure 2A,B).

Podocyte injury plays a critical role in glomerular damage [33]. Therefore, we investigated podocyte loss in response to HFD feeding using synaptopodin as a podocyte marker. The immunostaining results showed that synaptopodin was exclusively expressed in the glomeruli of the WKY, SHR, and SHR-SP rats (Figure 2C). Notably, the synaptopodin-positive areas were comparable among the WKY, SHR, and SHR-SP rats, regardless of whether they were fed a control diet or an HFD (Figure 2C). To assess synaptopodin levels further, we performed a Western blot analysis. The results showed a slight decrease in synaptopodin protein levels in the HFD-fed WKY rats compared to those in their control-diet-fed counterparts. In the SHR rats, the synaptopodin levels increased following the HFD treatment, whereas in SHR-SP, there was no significant difference between the control-diet-fed and HFD-fed groups (Figure 2D,E).

Tenascin-c (TNC) is an extracellular matrix glycoprotein that plays a pivotal role in tissue remodeling, inflammation, and fibrosis. Its upregulation is strongly associated with tubulointerstitial fibrosis, contributing to disease progression [34]. To determine whether the HFD influenced TNC expression, we examined the TNC protein levels in the WKY, SHR, and SHR-SP rats using immunostaining. In the WKY and SHR rats fed a control diet, the TNC expression was barely detectable (Figure 2F). However, in the HFD-fed WKY and SHR, the TNC levels were markedly increased. In contrast, the SHR-SP rats exhibited widespread TNC expression even under a control diet, suggesting a baseline predisposition towards fibrosis ((%/field): WKY control: 0.12 ± 0.048; WKY HFD: 0.262 ± 0.048; SHR control: 0.132 ± 0.043; SHR HFD: 0.753 ± 0.15; SHR-SP control: 1.984 ± 0.39; SHR-SP HFD: 1.464 ± 0.12 (*p* values: WKY control vs. WKY HFD: <0.005; SHR control vs. SHR HFD: <0.00005; SHR-SP control vs. SHR-SP HFD: <0.05; WKY control vs. SHR control: 0.73; WKY control vs. SHR-SP control: <0.00001; SHR control vs. SHR-SP control: <0.00001)) (Figure 2F,G). Interestingly, HFD treatment did not increase the TNC levels in the SHR-SP rats; instead, it led to a reduction, highlighting a potential strain-specific response (Figure 2F,G).

### 2.4. The Effects of a High-Fat Diet on Senescence-Related Changes in the Kidneys in Normotensive and Hypertensive Rat Models

**Figure 3:**
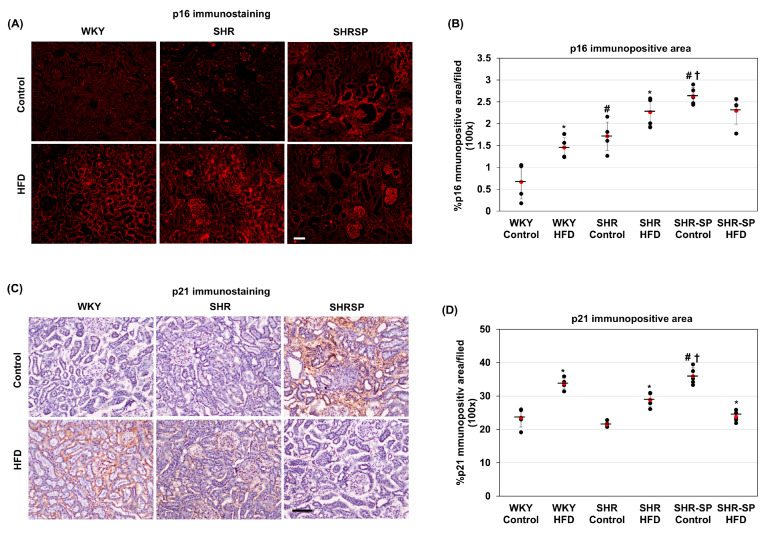
The effects of a high-fat diet on senescence in rat kidneys. To evaluate the impact of a high-fat diet on senescence in WKY, SHR, and SHR-SP rats, the levels of senescence markers, including p16 and p21, were assessed. Immunostaining photomicrographs depicting p16 (red) cells within the kidneys are shown in (**A**). p16-positive areas in the kidneys were quantified using ImageJ, expressed as the percent of the total area from photomicrographs captured at 100× magnification, and the averages of the immunopositive areas are shown in (**B**) (*n* = 5). (**C**) Representative immunostaining photomicrographs illustrating p21’s distribution in the kidneys are shown here. In (**D**), the averages of the immunopositive areas are presented (*n* = 5). The numerical data are presented as dot-plots with averages (marked by red dots with a line) and the ± SD. Statistical significance is indicated as follows: * *p* < 0.05 compared to control-diet-fed rats of the same strain; ^#^ *p* < 0.05 compared to control-diet-fed WKY rats. ^†^ *p* < 0.05 compared to control-diet-fed SHR rats. Bar = 100 µm.

A high-saturated-fat diet has been shown to induce cellular-senescence-related changes [35]. Senescence is shown to be associated with chronic inflammatory conditions and fibrosis [36]. Since fibrotic changes were observed in the WKY, SHR, and SHR-SP rats, we assessed the senescence levels by analyzing markers such as p16 and p21. Immunostaining for p16 revealed that it was undetectable in the cortical areas of the kidneys from the control-diet-fed WKY rats (Figure 3A). In SHR, the p16 levels were slightly but significantly increased compared to those in WKY, whereas this increase was greater for SHR-SP ((%/field): WKY control: 0.664 ±0.385; WKY HFD: 1.45 ± 0.222; SHR control: 1.71 ± 0.324; SHR HFD: 2.26 ± 0.299; SHR-SP control: 2.632 ± 0.196; SHR-SP HFD: 2.294 ± 0.305 (*p* values: WKY control vs. WKY HFD: <0.005; SHR control vs. SHR HFD: <0.05; SHR-SP control vs. SHR-SP HFD: <0.07; WKY control vs. SHR control: <0.005; WKY control vs. SHR-SP control: <0.00001; SHR control vs. SHR-SP control: <0.001)). When the rats were fed an HFD, the p16 levels were elevated in both the WKY and SHR rats compared to those in their control-diet-fed counterparts. However, in SHR-SP, the p16 levels did not increase with the HFD (Figure 3A,B).

**Figure 4:**
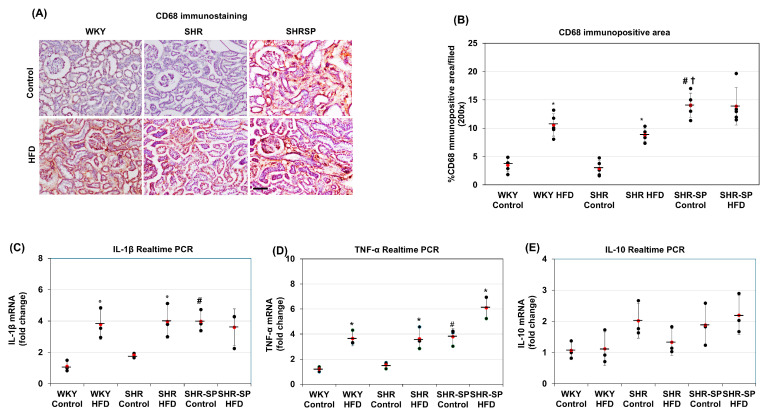
The effects of a high-fat diet on inflammatory conditions in rat kidneys. To evaluate the impact of a high-fat diet on inflammatory conditions in WKY, SHR, and SHR-SP rats, the levels of an inflammatory cell marker (CD68) were assessed. Immunostaining photomicrographs depicting CD68 cells within the kidneys are shown in (**A**). CD68-positive areas in the kidneys were quantified using ImageJ, expressed as the percentage of the total area from photomicrographs captured at 200× magnification, and the average immunopositive areas are shown in (**B**) (*n* = 5). Inflammatory changes were evaluated further by analyzing the mRNA of IL-1β, TNF-α, and IL-10 using real-time PCR. The average real-time PCR data for IL-1β (**C**), TNF-α (**D**), and IL-10 (**E**) are shown here (*n* = 3). The numerical data are presented as dot-plots with averages (marked by red dots with a line) and the ± SD. * *p* < 0.05 compared to control-diet-fed rats of the same strain; ^#^ *p* < 0.05 compared to WKY or SHR control rats; ^†^ *p* < 0.05 compared to control-diet-fed SHR rats. Bar = 100 µm.

Next, we examined the p21 levels in the kidneys through immunostaining. Similar to p16, p21 was undetectable in the cortical areas of the kidneys from the WKY rats fed a control diet (Figure 3C). In SHR, the p21 levels were similar to those in WKY and almost undetectable. But in the control-diet-fed SHR-SP rats, the p21 levels increased significantly compared to those in their WKY and SHR counterparts ((%/field): WKY control: 23.49 ±2.77; WKY HFD: 33.72 ± 1.63; SHR control: 21.48 ± 0.88; SHR HFD: 28.92 ± 2.04; SHR-SP control: 35.91 ± 2.52; SHR-SP HFD: 23.91 ± 1.599 (*p* values: WKY control vs. WKY HFD: <0.0005; SHR control vs. SHR HFD: <0.0001; SHR-SP control vs. SHR-SP HFD: <0.00005; WKY control vs. SHR control: 0.15; WKY control vs. SHR-SP control: <0.0001; SHR control vs. SHR-SP control: <0.00005)) (Figure 3C,D). In the SHR-SP rats, p21-positive cells were primarily located in the tubular interstitial space, with some cells in the tubular epithelium also showing positivity. When fed an HFD, p21-positive cells were found in the tubular epithelium and interstitial spaces of both the WKY and SHR rats (Figure 3C and Figure 4D). However, in SHR-SP, the p21 levels did not increase but rather decreased with the HFD (Figure 3D).

The Effects of a High-Fat Diet on Inflammatory Changes in the Kidneys in Normotensive and Hypertensive Rat Models. Inflammation is often associated with senescence-related cellular changes [36]. Since inflammation is also linked to fibrosis, we next investigated the accumulation of inflammatory cells in the kidneys through immunostaining and examined the mRNA expression of several inflammation-related cytokines using real-time PCR. Immunostaining revealed that in both the WKY and SHR rats fed a control diet, the number of CD68-positive macrophages was very low. However, in the SHR-SP rats on the same diet, the CD68-positive areas were significantly increased compared to those in the WKY and SHR rats ((%/field): WKY control: 3.47 ±1.16; WKY HFD: 10.57 ± 1.97; SHR control: 2.91 ± 1.34; SHR HFD: 8.84 ± 1.12; SHR-SP control: 14.07 ± 2.13; SHR-SP HFD: 13.88 ± 3.32 (*p* values: WKY control vs. WKY HFD: <0.0005; SHR control vs. SHR HFD: <0.0001; SHR-SP control vs. SHR-SP HFD: 0.915; WKY control vs. SHR control: 0.5; WKY control vs. SHR-SP control: <0.00005; SHR control vs. SHR-SP control: <0.00001)) (Figure 4A,B). These CD68-positive cells were primarily located in the tubular interstitial spaces. When the rats were fed the HFD, the CD68-positive areas increased in the WKY and SHR rats, reaching significantly higher levels than those in their control-fed counterparts. However, in the SHR-SP rats fed the HFD, the positive areas were similar to those in their control-diet-fed counterparts (Figure 4A,B).

Next, we analyzed the mRNA expression of inflammation-related cytokines using real-time PCR. In the control-diet-fed animals, the IL-1β and TNF-α mRNA levels were similar in the kidneys of the WKY and SHR rats but significantly elevated in the SHR-SP rats (Figure 4C,D). In the HFD-fed animals, the IL-1β and TNF-α mRNA levels in the kidneys of the WKY and SHR rats were significantly increased compared to those in the control-fed counterparts (IL-1β (fold change/calibrator): WKY control: 1.11 ± 0.35; WKY HFD: 3.78 ± 0.97; SHR control: 1.82 ± 0.14; SHR HFD: 3.97 ± 1.07; SHR-SP control: 3.98 ± 0.68; SHR-SP HFD: 3.6 ± 1.18 (*p* values: WKY control vs. WKY HFD: <0.05; SHR control vs. SHR HFD: <0.05; SHR-SP control vs. SHR-SP HFD: 0.68; WKY control vs. SHR control: <0.05; WKY control vs. SHR-SP control: <0.005; SHR control vs. SHR-SP control: <0.01); TNF-α (fold change/calibrator): WKY control: 1.21 ±0.2; WKY HFD: 3.64 ± 0.58; SHR control: 1.53 ± 0.26; SHR HFD: 3.62 ± 0.88; SHR-SP control: 3.79 ± 0.66; SHR-SP HFD: 6.1 ± 0.85 (*p* values: WKY control vs. WKY HFD: <0.005; SHR control vs. SHR HFD: <0.05; SHR-SP control vs. SHR-SP HFD: <0.05; WKY control vs. SHR control: 0.16; WKY control vs. SHR-SP control: <0.005; SHR control vs. SHR-SP control: <0.01)). However, in the SHR-SP rats fed an HFD, the IL-1β levels remained unchanged, while the TNF-α mRNA levels were significantly elevated compared to those in their control-diet-fed counterparts (Figure 4C,D). On the other hand, the IL-10 mRNA levels were similar among WKY, SHR, and SHR-SP rats fed a control diet, and the HFD did not significantly alter the IL-10 expression in any of the rat strains (IL-10 (fold change/calibrator): WKY control: 1.07 ± 0.28; WKY HFD: 1.12 ± 0.54; SHR control: 2.02 ± 0.56; SHR HFD: 1.33 ± 0.43; SHR-SP control: 3.98 ± 0.68; SHR-SP HFD: 3.6 ± 1.18 (*p* values: WKY control vs. WKY HFD: < 0.88; SHR control vs. SHR HFD: < 0.17; SHR-SP control vs. SHR-SP HFD: 0.59; WKY control vs. SHR control: <0.058; WKY control vs. SHR-SP control: 0.13; SHR control vs. SHR-SP control: 0.8)) (Figure 4E).

### 2.5. The Effects of a High-Fat Diet on Changes in Mesenchymal and Epithelial Markers in the Kidneys in Normotensive and Hypertensive Rat Models

**Figure 5:**
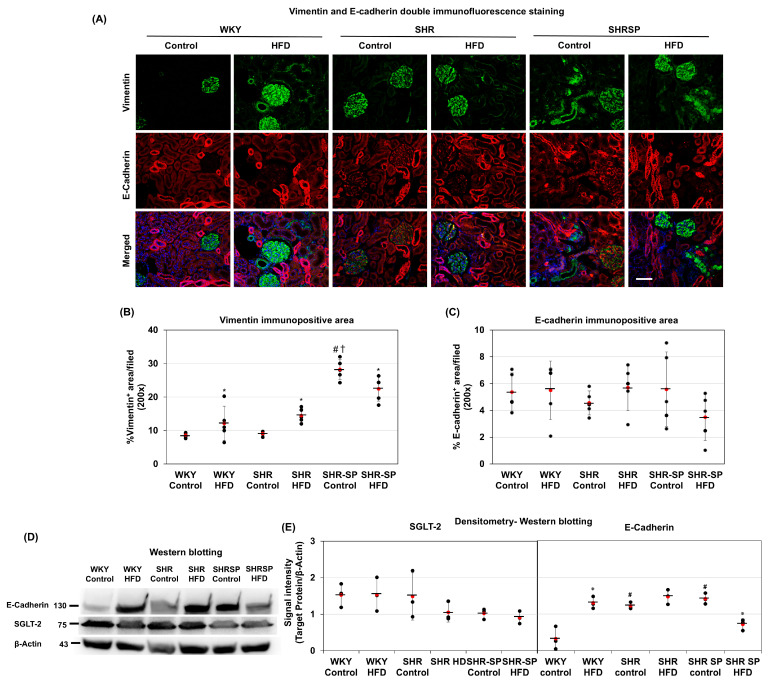
The effects of a high-fat diet on the epithelial-to-mesenchymal transition in rat kidneys. To assess the effects of a high-fat diet on the epithelial-to-mesenchymal transition (EMT) in WKY, SHR, and SHR-SP rats, the localization of the epithelial marker E-cadherin and the mesenchymal marker vimentin was analyzed using double immunofluorescence staining. Nuclear staining was performed using Hoechst (blue). (**A**) shows representative photomicrographs of double immunofluorescence staining for vimentin (green) and E-cadherin (red), along with merged images, for WKY, SHR, and SHR-SP rats fed either a control diet or a high-fat diet. The immunopositive areas of vimentin and E-cadherin in the kidney sections were quantified using ImageJ. The average quantified data for vimentin is presented in (**B**), and that for E-cadherin is shown in (**C**) (*n* = 5 each). A Western blot analysis was performed to evaluate the levels of E-cadherin and another epithelial marker, SGLT2, further. (**D**) Representative Western blot images for E-cadherin and SGLT2 are displayed, with β-actin used as a loading control. (**E**) A densitometric analysis of E-cadherin and SGLT2 Western blots was performed, and the averaged data are shown here (*n* = 3). The numerical data are presented as dot-plots with averages (marked by red dots with a line) and the ± SD. Statistical significance is denoted as follows: * *p* < 0.05 compared to control-diet-fed rats of the same strain. ^#^ *p* < 0.05 compared to control-diet-fed WKY rats. ^†^ *p* < 0.05 compared to control-diet-fed SHR rats. Bar = 100 µm.

The epithelial-to-mesenchymal transition (EMT) has been thought to play a crucial role in fibrosis, and inflammatory conditions further contribute to this process [37]. Therefore, we examined the effects of the HFD on the EMT in the kidneys. Vimentin was used as a mesenchymal marker, while E-cadherin served as an epithelial marker. Double immunofluorescence staining revealed that in the WKY and SHR rats fed a control diet, the vimentin expression was primarily confined to the glomerulus. However, in the SHR-SP rats, vimentin was also expressed in the tubular areas in addition to the glomerulus (Figure 5A). E-cadherin staining was restricted to the tubules in all three rat strains (Figure 5A). In the HFD-fed rats, vimentin was positive in the glomerulus and the tubular interstitial spaces of both the SHR and WKY rats. In the SHR-SP rats, vimentin was also strongly expressed in the interstitial areas. Merging the vimentin and E-cadherin signals showed no obvious co-localization of these markers in the tubular epithelial cells (Figure 5A).

We then quantified the vimentin and E-cadherin expression. The results showed that under a control diet, the vimentin levels were highest in the SHR-SP rats, while in the SHR rats, they were similar to those in the WKY rats ((%/field): WKY control: 8.58 ± 0.72; WKY HFD: 12.11 ± 5.13; SHR control: 9.08 ± 0.71; SHR HFD: 14.47 ± 2.12; SHR-SP control: 28.23 ± 2.98; SHR-SP HFD: 22.45 ± 3.66 (*p* values: WKY control vs. WKY HFD: 0.17; SHR control vs. SHR HFD: <0.001; SHR-SP control vs. SHR-SP HFD: <0.05; WKY control vs. SHR control: 0.3; WKY control vs. SHR-SP control: <0.000001; SHR control vs. SHR-SP control: <0.000001)) (Figure 5B). Upon HFD feeding, the vimentin levels increased in the WKY and SHR rats (Figure 5B). A further analysis of vimentin revealed that in the control-fed WKY and SHR rats, vimentin was nearly absent from the tubular interstitial spaces, whereas it was strongly expressed in the SHR-SP rats (Supplemental Figure S3A). In the HFD-fed rats, tubular interstitial vimentin levels increased in the WKY and SHR rats compared to those in their control-fed counterparts but remained unchanged in the SHR-SP rats (Supplemental Figure S3A).

Quantification of E-cadherin immunostaining revealed that the levels remained similar across all rat strains, regardless of diet (Figure 5C and Supplemental Figure S3B). However, the Western blot analysis showed that under a control diet, the E-cadherin levels were lower in the WKY and SHR rats compared to those in the SHR-SP rats ((E-cadherin/β-actin): WKY control: 0.33 ± 0.31; WKY HFD: 1.3 ± 0.16; SHR control: 1.22 ± 0.09; SHR HFD: 1.5 ± 0.2; SHR-SP control: 1.42 ± 0.15; SHR-SP HFD: 0.72 ± 0.15 (*p* values: WKY control vs. WKY HFD: <0.01; SHR control vs. SHR HFD: 0.12; SHR-SP control vs. SHR-SP HFD: <0.005; WKY control vs. SHR control: <0.01; WKY control vs. SHR-SP control: <0.01; SHR control vs. SHR-SP control: 0.13) (Figure 5D,E). Upon HFD feeding, the E-cadherin levels increased in the WKY and SHR rats but decreased in the SHR-SP rats (Figure 5D,E). Then, we examined another epithelial marker, SGLT-2, using Western blotting. The results showed that the HFD had no significant effect on the SGLT-2 expression in any of the rat strains (Figure 5D,E).

### 2.6. The Effects of a High-Fat Diet on TGF-β and TGF-βR2 in the Kidneys in Normotensive and Hypertensive Rat Models

**Figure 6:**
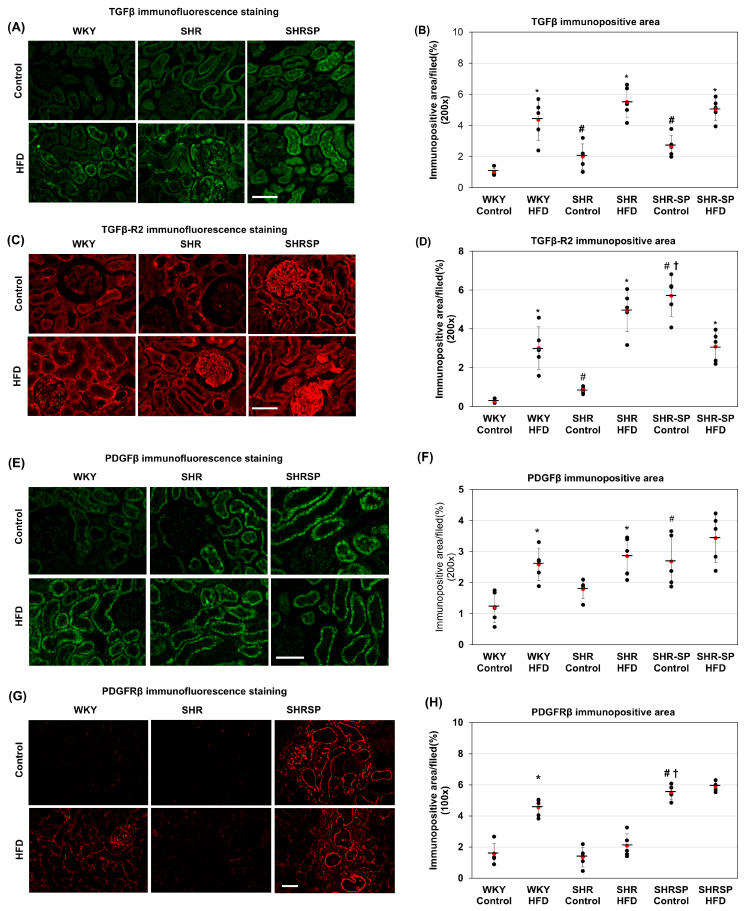
The effects of a high-fat diet on the levels of TGFβ and PDGFβ and their receptors in rat kidneys. To investigate the effects of a high-fat diet on TGFβ and TGFβ-R2 in the WKY, SHR, and SHR-SP rats, immunofluorescence staining was performed. (**A**) Representative photomicrographs of TGFβ-immunofluorescence-stained (green) cortical areas of the kidneys are shown here. (**B**) The immunopositive areas for TGFβ in the kidney sections were quantified using ImageJ, and the average quantified data is presented here (*n* = 5). (**C**) Representative photomicrographs of TGFβ-R2-immunofluorescence-stained (red) cortical areas of the kidneys are shown here. (**D**) The immunopositive areas for TGFβ-R2 in the kidney sections were quantified, and the average quantified data is presented here (*n* = 5). To investigate the effects of a high-fat diet on PDGFβ and PDGF-Rβ in WKY, SHR, and SHR-SP rats, immunofluorescence staining was performed. (**E**) Representative photomicrographs show immunofluorescence staining for PDGFβ (green) in the cortical kidney areas. (**F**) The immunopositive areas for PDGFβ in the kidney sections were quantified using ImageJ, and the average quantified data is presented here (*n* = 5). (**G**) Representative photomicrographs display the immunofluorescence staining for PDFRβ (red) in the cortical kidney areas. (**H**) The immunopositive areas for PDGFRβ in the kidney sections were quantified, and the average quantified data is presented here (*n* = 5). The numerical data are presented as dot-plots with averages (marked by red dots with a line) and the ± SD. Statistical significance is indicated as follows: * *p* < 0.05 compared to control-diet-fed rats of the same strain; ^#^ *p* < 0.05 compared to control-diet-fed WKY rats. ^†^ *p* < 0.05 compared to control-diet-fed SHR rats. Bar = 100 µm.

TGF-β signaling plays a crucial role in mesenchymal expansion and fibrosis [37]. Since we observed increased fibrosis and elevated mesenchymal markers, including vimentin, in the HFD-fed animals, we examined the expression of TGF-β and its receptor (TGF-βR2) in the kidneys. The immunostaining results showed that TGF-β expression was nearly absent in the kidneys of the WKY rats fed a control diet (Figure 6A). In contrast, the SHR rats fed a control diet exhibited a slight but significant increase in TGF-β levels, which was expressed mainly in the tubular areas. Similarly, the SHR-SP rats on a control diet showed further increased TGF-β expression compared to that in WKY rats ((%/field): WKY control: 1.01 ± 0.23; WKY HFD: 4.36 ± 1.31; SHR control: 2 ± 0.82; SHR HFD: 5.51 ± 1.01; SHR-SP control: 2.67 ± 0.7; SHR-SP HFD: 5.05 ± 0.72 (*p* values: WKY control vs. WKY HFD: <0.0005; SHR control vs. SHR HFD: <0.0005; SHR-SP control vs. SHR-SP HFD: 0.001; WKY control vs. SHR control: <0.05; WKY control vs. SHR-SP control: <0.001; SHR control vs. SHR-SP control: 0.2)) (Figure 6A,B). Upon HFD feeding, the TGF-β levels increased across all rat strains, with no significant differences among them (Figure 6A,B). In the HFD-fed rats, TGF-β was found to be expressed both in the tubular areas and in the glomeruli.

Next, we analyzed the TGF-βR2 expression in the kidneys. Immunostaining revealed that similar to TGF-β, the TGF-βR2 expression was nearly undetectable in the WKY rats on a control diet (Figure 6C). However, in the SHR rats, the TGF-βR2 levels were slightly but significantly increased compared to those in their WKY counterparts. The highest TGF-βR2 levels were observed in the SHR-SP rats on a control diet (Figure 6C,D). In WKY and SHR, TGF-βR2 was positive mainly in the tubular areas, whereas it was positive in both the glomeruli and in the tubular areas in SHR-SP. Following HFD feeding, the TGF-βR2 expression increased in the glomerular and tubular areas in the WKY and SHR rats, with no significant difference between the two strains ((%/field): WKY control: 0.26 ± 0.1; WKY HFD: 3.01 ± 1.1; SHR control: 0.86 ± 0.16; SHR HFD: 4.95 ± 1.1; SHR-SP control: 5.7 ± 1.07; SHR-SP HFD: 3.09 ± 0.78 (*p* values: WKY control vs. WKY HFD: <0.001; SHR control vs. SHR HFD: <0.00005; SHR-SP control vs. SHR-SP HFD: 0.005; WKY control vs. SHR control: <0.0001; WKY control vs. SHR-SP control: <0.000005; SHR control vs. SHR-SP control: <0.00001)) (Figure 6C,D). However, in the SHR-SP rats, the TGF-βR2 levels did not increase with the HFD; instead, they decreased compared to those in the control-fed SHR-SP rats. The Western blot analysis also confirmed that the TGF-βR2 levels increased significantly in the WKY rats fed an HFD compared to those in their control-fed counterparts (Supplemental Figure S4A,B).

### 2.7. The Effects of a High-Fat Diet on PDGFβ and PDGF-Rβ in the Kidneys of Normotensive and Hypertensive Rat Models

PDGF-β signaling plays a crucial role in fibrosis [38]. To investigate its involvement, we examined the expression of PDGF-β and its receptor (PDGF-Rβ) in the kidneys. The immunostaining results showed that the PDGF-β expression was nearly absent in the WKY rats fed a control diet. In the SHR rats, the PDGF-β levels were slightly increased, with their expression mainly localized to the tubular areas. The SHR-SP rats on a control diet exhibited an even higher PDGF-β expression compared to that in the WKY rats, with localization predominantly in the tubular regions ((%/field): WKY control: 1.22 ± 0.51; WKY HFD: 2.59 ± 0.52; SHR control: 1.8 ± 0.31; SHR HFD: 2.9 ± 0.63; SHR-SP control: 2.69 ± 0.84; SHR-SP HFD: 3.44 ± 0.79 (*p* values: WKY control vs. WKY HFD: <0.005; SHR control vs. SHR HFD: <0.01; SHR-SP control vs. SHR-SP HFD: 0.19; WKY control vs. SHR control: 0.0; WKY control vs. SHR-SP control: <0.05; SHR control vs. SHR-SP control: 0.057)) (Figure 6E,F). Following HFD feeding, the PDGF-β levels increased across all rat strains, although this increase was not statistically significant in SHR-SP (Figure 6F). In the HFD-fed animals, the PDGF-β expression remained localized to the tubular areas.

Next, we examined the PDGF-Rβ expression in the kidneys. Immunostaining revealed that the PDGF-Rβ expression was nearly undetectable in the WKY and SHR rats on a control diet (Figure 6G. However, in the SHR-SP rats on a control diet, the PDGF-Rβ expression was significantly higher, with strong localization in both the glomerular and tubular areas. Upon HFD feeding, the PDGF-Rβ expression increased in both the glomerular and tubular areas in the WKY rats ((%/field): WKY control: 1.56 ± 0.67; WKY HFD: 4.55 ± 0.57; SHR control: 1.35 ± 0.64; SHR HFD: 2.08 ± 0.77; SHR-SP control: 5.51 ± 0.47; SHR-SP HFD: 5.89 ± 0.29 (*p* values: WKY control vs. WKY HFD: <0.0001; SHR control vs. SHR HFD: 0.14; SHR-SP control vs. SHR-SP HFD: 0.16; WKY control vs. SHR control: 0.62; WKY control vs. SHR-SP control: <0.000005; SHR control vs. SHR-SP control: <0.000005)) (Figure 6G,H). However, in the SHR-SP rats, the PDGF-Rβ levels did not increase with an HFD. The Western blot analysis further confirmed that the PDGF-Rβ levels significantly increased in the WKY rats fed an HFD compared to those in their control-fed counterparts (Supplemental Figure S5A,B).

### 2.8. The Effects of a High-Fat Diet on TGFβ and PDGFβ Signaling in the Kidneys in Normotensive and Hypertensive Rat Models

**Figure 7:**
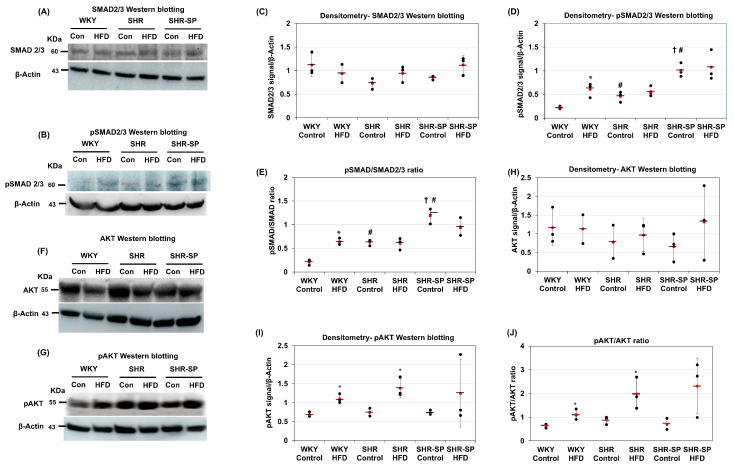
The effects of a high-fat diet on TGFβ and PDGFβ signaling in rat kidneys. To assess the effects of a high-fat diet on TGFβ and PDGFβ signaling, the transcription factors activated by TGFβ (SMAD2/3 phosphorylated into pSMAD2/3) and PDGFβ (AKT phosphorylated into pAKT) were analyzed through Western blotting. β-actin was used as a loading control, and the levels of target proteins were quantified through densitometry. Representative blots for SMAD2/3 and pSMAD2/3 are shown in (**A**) and (**B**), respectively. The average densitometric quantified data for SMAD2/3 (*n* = 3) and pSMAD2/3 (*n* = 3) are presented in (**C**) and (**D**), respectively. The ratio of pSMAD2/3 to SMAD2/3 is displayed in (**E**). Similarly, representative blots for AKT and pAKT are shown in (**F**) and (**G**), respectively. The average densitometric quantified data for AKT (*n* = 3) and pAKT (*n* = 3) are shown in (**H**) and (**I**), respectively. The ratio of pAKT to AKT is displayed in (**J**). The numerical data are presented as dot-plots with averages (marked by red dots with a line) and the ± SD. Statistical significance is indicated as follows: * *p* < 0.05 compared to control-diet-fed rats of the same strain; ^#^ *p* < 0.05 compared to control-diet-fed WKY rats. ^†^ *p* < 0.05 compared to control-diet-fed SHR rats.

Since TGF-β, PDGF-β, and their receptors were affected by the HFD, we next examined the activation of TGF-β and PDGF-β signaling in the rat kidneys. The Western blotting results showed that the levels of the TGF-β-dependent transcription factor SMAD2/3 varied among the rat strains. The WKY rats fed a control diet exhibited higher SMAD2/3 levels than those in their SHR and SHR-SP counterparts, though these differences were not statistically significant (SMAD2/3 (fold change/calibrator): WKY control: 1.12 ± 0.24; WKY HFD: 0.94 ± 0.19; SHR control: 0.73 ± 0.11; SHR HFD: 0.94 ± 0.17; SHR-SP control: 0.85 ± 0.04; SHR-SP HFD: 1.12 ± 0.2 (*p* values: WKY control vs. WKY HFD = 0.36; SHR control vs. SHR HFD = 0.14; SHR-SP control vs. SHR-SP HFD = 0.08; WKY control vs. SHR control = 0.06; WKY control vs. SHR-SP control = 0.13; SHR control vs. SHR-SP control = 0.15); pSMAD2/3 (fold change/calibrator): WKY control: 0.22 ± 0.03; WKY HFD: 0.61 ± 0.15; SHR control: 0.46 ± 0.11; SHR HFD: 0.56 ± 0.11; SHR-SP control: 1.01 ± 0.14; SHR-SP HFD: 1.08 ± 0.32 (*p* values: WKY control vs. WKY HFD: <0.05; SHR control vs. SHR HFD: 0.32; SHR-SP control vs. SHR-SP HFD: 0.76; WKY control vs. SHR control: <0.05; WKY control vs. SHR-SP control: <0.001; SHR control vs. SHR-SP control: <0.01); pSMAD/SMAD: WKY control: 0.2 ±0.06; WKY HFD: 0.64 ± 0.07; SHR control: 0.63 ± 0.06; SHR HFD: 0.6 ± 0.13; SHR-SP control: 1.18 ± 0.16; SHR-SP HFD: 0.96 ± 0.19 (*p* values: WKY control vs. WKY HFD: <0.005; SHR control vs. SHR HFD: 0.8; SHR-SP control vs. SHR-SP HFD: 0.18; WKY control vs. SHR control: <0.005; WKY control vs. SHR-SP control: <0.001; SHR control vs. SHR-SP control: <0.005)) (Figure 7A,C). In the HFD-fed groups, these differences were minimal. However, phosphorylated SMAD2/3 levels showed a distinct pattern. They were lowest in the kidneys of the WKY rats fed a control diet and highest in the kidneys of the SHR-SP rats on the same diet (Figure 7B,D). In the SHR rats fed a control diet, the phosphorylated SMAD2/3 levels were higher than those in the WKY rats but lower than those in the SHR-SP rats. In the HFD-fed groups, phosphorylated SMAD2/3 levels increased only in the WKY rats, with no significant changes observed in the SHR and SHR-SP rats (Figure 7D). The ratio of phosphorylated to total SMAD2/3 followed a similar trend. It was lowest in the WKY rats fed a control diet and highest in the SHR-SP rats on the same diet. In the SHR rats, this ratio was higher than that in the WKY rats but lower than that in the SHR-SP rats under the control diet. Among the HFD-fed groups, an increase in this ratio was observed only in the WKY rats (Figure 7E).

For PDGF-β signaling, we examined the activation of the PDGF-β-dependent transcription factor AKT. The Western blot analysis showed that the total AKT levels remained similar across the WKY, SHR, and SHR-SP rats, regardless of whether they were fed a control diet or an HFD (AKT (fold change/calibrator): WKY control: 1.17 ± 0.48; WKY HFD: 1.13 ± 0.38; SHR control: 0.78 ± 0.44; SHR HFD: 0.97 ± 0.44; SHR-SP control: 0.66 ± 0.38; SHR-SP HFD: 1.32 ± 0.998 (*p* values: WKY control vs. WKY HFD = 0.7; SHR control vs. SHR HFD = 0.42; SHR-SP control vs. SHR-SP HFD = 0.55; WKY control vs. SHR control = 0.4; WKY control vs. SHR-SP control = 0.41; SHR control vs. SHR-SP control = 0.93); pAKT (fold change/calibrator): WKY control: 0.68 ± 0.07; WKY HFD: 1.1 ± 0.12; SHR control: 0.75 ± 0.1; SHR HFD: 1.38 ± 0.26; SHR-SP control: 0.73 ± 0.06; SHR-SP HFD: 1.24 ± 0.89 (*p* values: WKY control vs. WKY HFD: <0.01; SHR control vs. SHR HFD: <0.05; SHR-SP control vs. SHR-SP HFD: 0.38; WKY control vs. SHR control: 0.42; WKY control vs. SHR-SP control: 0.4; SHR control vs. SHR-SP control: 0.85); pAKT/AKT: WKY control: 0.63 ±0.08; WKY HFD: 1.1 ± 0.22; SHR control: 0.88 ± 0.16; SHR HFD: 1.98 ± 0.66; SHR-SP control: 0.71 ± 0.23; SHR-SP HFD: 2.3 ± 1.17 (*p* values: WKY control vs. WKY HFD: <0.05; SHR control vs. SHR HFD: <0.05; SHR-SP control vs. SHR-SP HFD: 0.08; WKY control vs. SHR control: 0.08; WKY control vs. SHR-SP control: 0.6; SHR control vs. SHR-SP control: 0.37)) (Figure 7F,H). However, phosphorylated AKT levels showed a different pattern. Among the control-fed rats, the phosphorylated AKT levels were comparable across all three strains. In contrast, HFD feeding led to an increase in phosphorylated AKT levels in the WKY, SHR, and SHR-SP rats compared to those in their control-fed counterparts (Figure 7G,I). However, this increase was statistically significant only in the kidneys from the WKY and SHR rats, while the SHR-SP rats showed no significant change. The ratio of phosphorylated to total AKT followed a similar trend. Among the HFD-fed groups, this ratio increased in all three strains, with statistical significance observed only in the WKY and SHR rats (Figure 7J).

### 2.9. A Gene Ontology Analysis of Kidney-Fibrosis-Related Genes in the SHR-SP Rats

**Figure 8:**
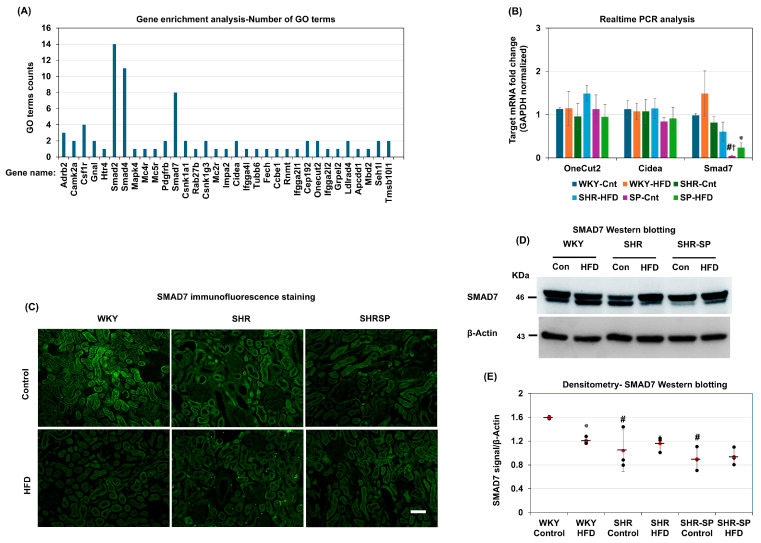
Gene Ontology analysis of kidney-fibrosis-related genes in SHR-SP rats. To investigate the fibrotic process in SHR-SP rats, a GO analysis of a DNA fragment from chromosome 18 was performed using the DAVID functional annotation tool. GO terms related to cell signaling, receptor functions, extracellular matrix regulation, and fibrosis were curated, and the genes enriched in these GO terms were identified. The GO term counts for each gene are presented in (**A**). Genes associated with the GO term “Negative regulation of TGF-beta receptor signaling pathways (GO:0030512)” were analyzed further using real-time PCR, and the average results are shown in (**B**) (*n* = 3). SMAD7, an inhibitory SMAD enriched in this GO term, was examined via immunostaining. Representative immunostaining photomicrographs of the cortical kidney tissues from WKY, SHR, and SHR-SP rats fed either a control diet or a high-fat diet (HFD) are shown in (**C**). To quantify the SMAD7 levels in the kidneys further, a Western blot analysis was conducted. A representative SMAD7 blot is shown in (**D**), with β-actin used as a loading control. The SMAD7 blot signals were quantified through densitometry, and the quantified data are displayed in (**E**) (*n* = 3). Statistical significance is indicated as follows: * *p* < 0.05 compared to control-diet-fed rats of the same strain; ^#^ *p* < 0.05 compared to control-diet-fed WKY rats. ^†^ *p* < 0.05 compared to control-diet-fed SHR rats. Bar = 100 µm.

Since the SHR-SP rats exhibited extensive kidney fibrosis, which was largely unaffected by a high-fat diet (HFD), we next investigated the underlying cause of this fibrosis. Previous studies have shown that a DNA fragment on chromosome 18 in SHR-SP rats (SHR.SHR-SP-(D18Rat73-D18Rat11)/Izm) is associated with kidney fibrosis independently of hypertension [39]. An analysis of this DNA fragment identified 328 genes, including 144 protein-coding genes (Supplemental Materials). To gain insight into their functional roles, we performed a Gene Ontology (GO) analysis using the DAVID functional annotation tool. The GO analysis revealed enrichment in 43 biological process (BP) terms, 22 cellular component (CC) terms, and 16 molecular function (MF) terms (Supplemental Materials). We then curated GO terms related to cellular signaling, enzyme activity, receptor activity, extracellular matrix regulation, and fibrosis (Supplemental Materials). Next, we quantified how many GO terms each gene was enriched in. The results highlighted key genes involved in TGF-β signaling, including SMAD2 (enriched in 14 GO terms), SMAD4 (11 GO terms), and SMAD7 (8 GO terms) (Figure 8A). One particularly relevant GO term, negative regulation of the TGF-β receptor signaling pathway (GO:0030512), which includes SMAD7, Cidea, Onecut2, and Ldlrad4, was analyzed further (Supplemental Materials). Real-time PCR results showed that the SMAD7 mRNA expression was significantly lower in the kidneys of the SHR-SP rats fed a control diet compared to that in the WKY and SHR rats on the control diet (Figure 8B). In the HFD-fed WKY and SHR rats, SMAD7 mRNA levels remained unchanged compared to those in their control-fed counterparts. However, in the SHR-SP rats, SMAD7 mRNA levels were slightly but significantly increased in the HFD group compared to those in the control diet group (SMAD7 (fold change/calibrator): WKY control: 0.98 ± 0.05; WKY HFD: 1.49 ± 0.52; SHR control: 0.81 ± 0.14; SHR HFD: 0.61 ± 0.22; SHR-SP control: 0.04 ± 0.02; SHR-SP HFD: 0.24 ± 0.11 (*p* values: WKY control vs. WKY HFD: 0.18; SHR control vs. SHR HFD: 0.25; SHR-SP control vs. SHR-SP HFD: <0.05; WKY control vs. SHR control: 0.09; WKY control vs. SHR-SP control: <0.0000005; SHR control vs. SHR-SP control: <0.001)) (Figure 8B).

The immunostaining results confirmed these findings further. The SMAD7 levels were high in the kidneys of the WKY rats fed a control diet but were reduced in the SHR rats on the same diet (Figure 8C). In the SHR-SP rats, the SMAD7 levels were even lower. Moreover, in the WKY and SHR rats fed an HFD, the SMAD7 levels decreased compared to those in their control diet counterparts. However, in the SHR-SP rats, this reduction was not observed (Figure 8C). Western blotting also showed that SMAD7 protein levels were significantly lower in the SHR-SP rats on a control diet compared to those in the WKY and SHR rats, and HFD feeding decreased them only in the WKY rats (SMAD7/β-actin: WKY control: 1.6 ± 0.02; WKY HFD: 1.21 ± 0.06; SHR control: 1.04 ± 0.35; SHR HFD: 1.16 ± 0.13; SHR-SP control: 0.9 ± 0.2; SHR-SP HFD: 0.94 ± 0.15 (*p* values: WKY control vs. WKY HFD: <0.005; SHR control vs. SHR HFD: 0.6; SHR-SP control vs. SHR-SP HFD: 0.8; WKY control vs. SHR control: 0.0504; WKY control vs. SHR-SP control: <0.005; SHR control vs. SHR-SP control: 0.58) (Figure 8D,E).

## 3. Discussion

Hypertension and obesity are well-established risk factors for CKD [9,40]. They are the two most prevalent health conditions worldwide. Obesity in particular has increased at an alarming rate due to the widespread consumption of high-fat diets like the Western diet [22]. Given that hypertension and obesity frequently coexist, understanding their interaction in CKD progression is critical. Our study examined the impact and interactions of moderate hypertension (as modeled using SHR rats) and severe hypertension (modeled using SHR-SP rats) under an HFD in the context of kidney fibrosis. We found that SHR rats developed mild kidney fibrosis, whereas SHR-SP rats exhibited severe and widespread fibrosis. These findings suggest that the mechanisms underlying kidney fibrosis differ between moderate and severe hypertension conditions. Furthermore, our HFD experiments demonstrated that these two hypertensive models responded differently to dietary fat, likely due to distinct molecular mechanisms and genetic backgrounds [41]. In both the WKY and SHR rats, the HFD exacerbated kidney damage to a similar extent, suggesting that HFD-induced fibrosis shares common pathways in normotensive and moderate hypertensive conditions. These findings indicate the importance of addressing obesity and dietary factors in the early stages of CKD to improve disease management.

The histological analysis revealed the presence of RBCs in the kidney parenchyma of the SHR-SP rats on a control diet, as well as in the WKY, SHR and SHR-SP rats fed an HFD. Since SHR-SP rats develop severe hypertension [42], this suggests that RBC infiltration results from hypertension-induced vascular injury. Severe hypertension conditions like malignant hypertension can lead to fibrinoid necrosis, causing significant vascular damage, ischemia, and RBC leakage [43]. An HFD is also known to impair vascular integrity through inflammation and lipid deposition, which may contribute to RBC accumulation in the kidneys [23,24,25]. The resulting vascular damage can trigger inflammation, hypoxia, and fibrosis, exacerbating kidney injury further. Indeed, evidence of kidney injury like fibrosis was seen both in severe hypertension and HFD conditions. However, the HFD did not increase the fibrosis in severely hypertensive SHR-SP rats. One possible explanation is that severe hypertension alone induces sufficient fibrosis, leaving no additional effect for the HFD. Alternatively, hypertension may initiate a fibrotic process, and the contribution of the HFD to which requires more time to become apparent. As our model was designed to assess early pathological changes, later synergistic or additive effects between hypertension and the HFD may have been missed due to this time limitation. Another possibility is that the mechanisms of kidney damage in SHR-SP may differ from those in WKY and SHR rats [39]. Notably, one study reported that a DNA fragment on chromosome 18 in SHR-SP can cause kidney fibrosis independent of hypertension [40]. Additionally, previous research found that HFD-fed SHR-SP rats experienced a decrease in blood pressure and reduced stroke occurrence [44]. These findings suggest that the interaction between the HFD and the cardiovascular system in SHR-SP may differ from that in WKY and SHR due to genetic differences. Further studies are needed to clarify how SHR-SP genetics influence the responses to an HFD and how these interactions differ from those in WKY and SHR rats.

Since vascular injury was observed in severe hypertension and in the animals fed an HFD, we examined whether other kidney components were also affected. Klotho, a multifunctional protein expressed in the kidneys, brain, and parathyroid glands, plays a critical role in regulating renal function, phosphate metabolism, aging, oxidative stress, and inflammation [32,45]. Its expression is known to decrease in cases of kidney tubular injury. Clinically, serum Klotho levels are reduced in both hypertension and obesity [46,47]. Our findings showed that the Klotho levels were decreased in the tubular cells of the kidneys in the SHR and SHR-SP rats on a control diet, as well as in the HFD-fed WKY rats. In the SHR and SHR-SP rats, the HFD did not reduce the Klotho expression further, suggesting that the regulation of Klotho is complex and may differ between HFD-induced and hypertension-induced conditions. However, since the rats were fed an HFD for only two months, this duration may have been insufficient to fully observe its inhibitory effects on Klotho expression under hypertensive conditions. The observed reduction in Klotho is likely driven by oxidative stress and inflammatory cytokines, both of which are elevated in hypertension and under HFD conditions [48,49]. However, in the SHR rats on a control diet, the TNF-α levels were similar to those in WKY rats, suggesting that oxidative stress, rather than inflammatory cytokines, is the primary driver of the reduction in Klotho in hypertensive conditions. Klotho inhibits fibrotic signaling pathways (e.g., TGF-β) and proinflammatory responses, while inflammation can in turn suppress Klotho expression [48,50]. Hence, due to this reciprocal relationship, a vicious cycle may establish that promotes fibrosis in affected organs such as the kidneys.

Hypertension- and HFD-induced kidney fibrosis is a complex process that affects both the glomerular and tubulointerstitial compartments [51,52]. These fibrotic changes are typically classified as tubulointerstitial fibrosis (TIF) or glomerulosclerosis, both of which are hallmarks of CKD and major contributors to renal dysfunction. Tenascin-C (TNC), an extracellular matrix (ECM) glycoprotein, plays a crucial role in the development of TIF, while ECM deposition by activated mesangial cells is critical in glomerulosclerosis [34,52]. Upon activation, the mesangial cells acquire a mesenchymal phenotype, secrete excessive ECM, and are marked by increased expression of SMC α-actin [53]. In the tubular interstitial areas of the kidneys from the SHR-SP mice, a significantly higher TNC expression was observed compared to that in WKY and SHR. In contrast, moderate hypertension (as seen in SHR) resulted in only a minor change in TNC expression. The HFD elevated the TNC levels in both the WKY and SHR rats, with a greater increase observed in the latter. However, the TNC levels in the SHR HFD-fed rats did not reach those observed in SHR-SP. Interestingly, the HFD did not elevate the TNC expression further in the SHR-SP rats. Similar to TNC, the SHR-SP rats exhibited a higher abundance of SMC-α-actin-positive cells in the glomeruli compared to that seen in WKY and SHR. These findings suggest that severe hypertension is a stronger driver of both glomerulosclerosis and TIF than an HFD. Such pronounced TNC expression and mesenchymal expansion in SHR-SP may result from vascular injury caused by sustained high blood pressure, leading to inflammatory and degenerative responses [54]. Although SHR rats also exhibit hypertension, its severity is lower than that in SHR-SP rats, indicating that renal autoregulation is important and only severe hypertension may trigger significant kidney injury. Indeed, renal autoregulation is shown to be intact in SHR and can prevent hypertension-induced kidney damage [55,56]. It is also possible that differential regulation of the signaling pathways occurs under moderate versus severe hypertensive conditions. Indeed, previous studies have shown that angiotensin signaling is elevated in SHR-SP [57], which may enhance TGF-β signaling and promote fibrosis. While we did not assess angiotensin pathway activation in our models, the expression pattern for TGFR-β closely resembled that for TNC and SMC α-actin, aligning with fibrotic changes under both moderate and severe hypertension. Notably, the effects of the HFD on TNC and SMC α-actin expression did not appear to correlate with hypertension. The HFD did not exert significant additive effects in moderately hypertensive rats compared to those in normotensive rats and failed to increase their expression in the presence of severe hypertension. This suggests that an HFD and hypertension may contribute to kidney fibrosis through independent mechanisms, at least in the early stages of exposure. In the context of an HFD, factors such as oxidative stress, inflammatory signaling, TGF-β-mediated fibrosis, and podocyte lipotoxicity are likely contributors to kidney damage. However, we did not observe a reduction in the podocyte marker synaptopodin, suggesting that short-term HFD exposure had not yet caused deleterious podocyte injury. Therefore, mesenchymal expansion may have been the primary driver of glomerular fibrosis during the early stages. Although the HFD increased the TNC and SMC α-actin expression in normotensive and moderately hypertensive rats, their levels did not reach those observed under severe hypertension. This reinforces the conclusion that severe hypertension has greater potential to induce kidney fibrosis than an HFD. Nevertheless, the HFD still promoted TNC expression in the tubulointerstitial compartments and mesenchymal expansion in the glomeruli under normotensive and moderately hypertensive conditions, underscoring its role in the development of kidney fibrosis.

Cellular senescence and the resulting inflammation are considered some of the main contributors to the development and progression of fibrosis [36]. Given that both hypertension and an HFD influence the fibrotic process in complex ways, we examined how senescence is regulated under these conditions. We found that p16- and p21-positive senescent cells were minimal in the WKY rats but significantly increased in the glomeruli and tubular epithelial cells of the SHR-SP rats, with at least p16 elevated in the SHR rats as well. These findings suggest that hypertension plays a regulatory role in the induction of senescence. Importantly, the p21 expression was increased by the HFD in both the WKY and SHR rats, indicating that an HFD is also a strong inducer of senescence in the kidneys. Previous studies have shown that p16 and p21 drive distinct repertoires of senescence-associated secretory phenotype (SASP) proteins [58,59]. As a result, they influence neighboring cells through different signaling pathways. Although we did not analyze the SASP expression in detail in the context of hypertension and an HFD, the distribution of p21-positive areas closely matched the expression pattern of inflammatory cytokines, highlighting the role of senescence in modulating inflammatory responses under these conditions. Furthermore, TGF-β, a key SASP component [60], was differentially regulated by hypertension and the HFD, suggesting distinct senescence-related effects in each condition. Although senescence is known to promote the epithelial-to-mesenchymal transition (EMT) as a mechanism to drive fibrosis [61], we did not observe the EMT in response to hypertension, the HFD, or their combination. Therefore, the mesenchymal expansion observed in our study was likely driven by senescence-associated inflammation and fibrotic signaling rather than the EMT.

The TGF-β signaling pathway plays central roles in the development of kidney fibrosis [62]. TGF-β promotes fibroblast activation, myofibroblast differentiation, and collagen deposition through both SMAD-dependent and non-SMAD pathways [63]. Elevated levels of TGF-β receptors (TGF-Rβ) were observed in the SHR-SP rats on a control diet. The HFD had minimal additional impact on the TGF-Rβ levels in the SHR-SP rats, likely due to the already high baseline activation of TGF-β signaling associated with severe hypertension. In contrast, the TGF-Rβ levels were similar between the SHR and SHR-SP rats on a control diet but were increased by the HFD in all rat strains. Additionally, the expression of the inhibitory SMAD protein, SMAD7, appeared to be low in both the SHR and SHR-SP rats. While elevated levels of TGF-β and its receptors have been well documented in the context of hypertension and an HFD [64,65], the regulatory mechanisms controlling the inhibitory components of this signaling pathway are less well understood. A previous study identified a DNA fragment on chromosome 18 in SHR-SP rats that contributes to kidney fibrosis independently of hypertension [39]. This region includes genes encoding both activator and inhibitor SMADs, suggesting that SMAD regulation may be crucial for kidney fibrosis. Given that SMAD7 expression was reduced at both the mRNA and protein levels in the SHR-SP rats, at the protein level in the SHR rats on a control diet, and in the WKY rats following the HFD, the regulation of inhibitory components of the TGF-β pathway, particularly SMAD7, might be an important factor in the development of kidney fibrosis. The combination of increased TGF-β signaling and decreased inhibitory signals, such as SMAD7, may contribute to overall augmentation of the TGF-β activity under hypertensive and HFD conditions. This imbalance between signaling activation and inhibition could be the underlying cause of the observed increase in SMAD2/3 activation. However, the detailed mechanisms regarding SMAD7 expression and its role in kidney fibrosis in the context of hypertension and an HFD need to be thoroughly investigated.

In our study, similarly to TGF-Rβ, elevated levels of PDGF-Rβ were observed in the kidneys of the SHR-SP rats. In contrast, the PDGF-Rβ levels were comparable in the kidneys of the WKY and SHR rats. An HFD-induced increase in PDGF-Rβ was seen only in the WKY rats, not in SHR or SHR-SP. These findings suggest that with respect to hypertension and an HFD, the regulation of PDGF-Rβ expression is complex. The DNA fragment associated with kidney fibrosis in SHR-SP rats contains the PDGF-Rβ gene. Therefore, it is possible that increased PDGF-Rβ levels are partly due to genetic differences among the rat strains. Notably, phosphorylation of AKT, a downstream signaling molecule of PDGF-β, was similarly elevated in the WKY, SHR, and SHR-SP rats, indicating that PDGF-β signaling is primarily influenced by HFD, with only a minor contribution from hypertension. Nonetheless, further investigation is required to fully understand the role of PDGF-β signaling in the context of hypertension and high-fat-diet exposure.

## 4. Materials and Methods

### 4.1. Animals

Male spontaneously hypertensive rats (SHR) and spontaneously hypertensive stroke-prone rats (SHR/SP) were utilized as models of hypertension, while Wistar Kyoto (WKY) rats served as the normotensive controls. The animals had free access to food and water and were housed under controlled conditions, including a temperature of 23 ± 2 °C and a 12 h light/dark cycle. All of the experimental procedures adhered to the ARRIVE (Animal Research: Reporting of In Vivo Experiments) guidelines and the regulations of the Institute of Experimental Animals, Shimane University. The experimental protocols were reviewed and approved by the Ethical Committee of Shimane University (approval number: IZ2-96).

### 4.2. The Experimental Design

To examine the effects of a high-saturated-fat diet on kidney fibrosis, 12-week-old WKY, SHR, and SHR-SP rats were fed either a normal diet (ND) or a high-fat diet (HFD), in which 60% of the caloric content was derived from lard. The study design included six experimental groups, each comprising eight animals: (a) WKY-ND (*n* = 8), WKY-HFD (*n* = 8), SHR-ND (*n* = 8), SHR-HFD (*n* = 8), SHR-SP-ND (*n* = 8), and SHR-SP-HFD (*n* = 8). The kidneys from three rats per group were used for the real-time PCR and Western blot analyses, while the kidneys from five rats per group were allocated to the histological and immunostaining studies. The rats were maintained on their designated diets for eight weeks, during which their body weight was measured weekly. At the end of the feeding period, the animals were sacrificed for further analysis.

### 4.3. Tissue Preparation for Staining

For staining, 5 rats from each group were used. At the end of the feeding period, the rats were deeply anesthetized with isoflurane (Pfizer, New York, NY, USA) and transcardially perfused with normal saline. This was followed by perfusion with 4% paraformaldehyde in 0.1 M phosphate buffer (pH = 7.4) to fix the tissues. The kidneys were then removed, post-fixed in the same fixative overnight, and cryoprotected in 30% sucrose in 0.1 M phosphate buffer (pH = 7.4) for 24 h. After cryoprotection, 2 mm transverse sections were obtained around the hilar region, embedded into TissueTek OCT compound, and frozen using dry ice. For staining, 4–8 µm thin tissue slices were prepared using a cryostat, mounted onto glass slides, and stored at −20 °C until further use.

### 4.4. Hematoxylin and Eosin (H&E) Staining

Kidney tissue sections (8 µm thick) mounted onto glass slides were used for H&E staining to evaluate histological changes. The sections were first stained with Mayer’s hematoxylin (Wako, Monza, Italy) for 3 min to highlight the nuclear components. Excess hematoxylin was removed by rinsing the slides under running tap water for 20 min. Subsequently, Eosin Y solution (0.5% in water, Wako) was applied to the tissue for 2–3 min to stain the cytoplasmic components. The slides were briefly rinsed in distilled water, dehydrated through a graded ethanol series (70%, 80%, 90%, and two changes of 100%, 5 min each), and cleared with 2 changes of xylene for 5 min. Finally, the sections were mounted with a synthetic resin-based mounting medium (Thermo Fisher Scientific, Waltham, MA, USA) and covered with coverslips. The stained slides were examined and imaged using a Nikon light and fluorescent microscope system to assess histopathological features.

### 4.5. Periodic Acid–Schiff (PAS) Staining

Kidney tissue sections (4–8 µm thick) mounted onto glass slides were used for PAS staining to evaluate the basement membranes and glycogen content. After rinsing them in distilled water, the sections were treated with 0.5% periodic acid solution (Sigma-Aldrich, Waltham, MA, USA) for 10 min at room temperature. The slides were then washed thoroughly in distilled water and stained with Schiff’s reagent (Sigma-Aldrich) for 15 min. Excess reagent was removed by washing the slides in running tap water for 5 min, followed by counterstaining with Mayer’s hematoxylin (Wako) for 2 min to highlight the nuclear components. The slides were rinsed under running tap water, dehydrated using a graded ethanol series (70%, 80% 90%, and 2 changes of 100%, 5 min each), and cleared in 2 changes of xylene for 5 min. Finally, the sections were mounted with a synthetic resin-based mounting medium (Thermo Fisher Scientific) and covered with coverslips.

### 4.6. Azan Staining

Kidney tissue sections (4 µm thick) mounted onto glass slides were stained using the Azan staining method to evaluate fibrosis and connective tissue deposition. The tissue sections were stained with azocarmine solution (Sigma-Aldrich) for 10–15 min at room temperature to label the nuclei and cytoplasm. Excess azocarmine was removed by differentiating the sections in 1% acetic acid for 2 min, followed by rinsing them in distilled water. The slides were then treated with phosphotungstic acid solution (5%, Sigma-Aldrich) for 5–10 min to selectively remove azocarmine from the connective tissues while retaining the staining in the other structures. After rinsing them in distilled water, the sections were stained with aniline blue solution (Sigma-Aldrich) for 5–10 min. Finally, the slides were rinsed briefly in distilled water, dehydrated using a graded ethanol series (70%, 80%, 90%, and 2 changes of 100%, 5 min each), cleared in 2 changes of xylene for 5 min, mounted with a synthetic resin-based mounting medium (e.g., DPX, Thermo Fisher Scientific), and covered with coverslips.

### 4.7. Immunostaining

Kidney tissue sections (8 µm thick) mounted onto glass slides were used for immunostaining, following the previously described methods [66]. Briefly, endogenous peroxidase activity was quenched using 0.3% H_2_O_2_, and the sections were incubated in a blocking solution containing 10% normal goat or horse serum and 0.2% Triton X-100 in PBS. The sections were then incubated with primary antibodies overnight at 4 °C. For immunofluorescence staining, the tissue sections were subsequently incubated with Texas-Red-conjugated or FITC-conjugated species-specific secondary antibodies, and the nuclei were counterstained with Hoechst. For light microscopy, the sections were incubated with biotin-conjugated species-specific secondary antibodies (1:100, Vector, Ingold Road, CA, USA), followed by an avidin–biotin–peroxidase complex (ABC, Vector). The immune reaction products were visualized using 3,3′-diaminobenzidine (DAB, Sigma, St. Louis, MO, USA), and the tissues were counterstained with hematoxylin. When antigen retrieval was necessary, the slides were incubated in 10 mM citrate buffer (pH = 6.0) under boiling conditions for 20 min prior to quenching the endogenous peroxidase activity.

### 4.8. Quantification of Staining

For quantification of the histologically stained or immunostained tissues, two tissue slices approximately 2 mm apart around the hilum were used. The tissue sections were examined using a light and fluorescence microscope system (NIKON, ECLIPSE E600, Tokyo, Japan). In each slice, at least five images from the cortical areas were randomly captured at ×20 magnification. The histologically stained or immunopositive areas were quantified using ImageJ software (version ij154-win-java8), and the average of all 10 photomicrographs from 2 tissue slices was calculated. This average was considered the immunopositive area or the histologically stained area for each rat.

### 4.9. Total RNA Isolation, Reverse Transcription, and Quantitative Real-Time PCR

Total RNA was isolated from the renal cortical areas of the rats (*n* = 3 per group) using Trizol reagent (Invitrogen, Carlsbad, CA, USA) following the manufacturer’s instructions. The quality and concentration of the RNA were assessed using a spectrophotometer (NanoDrop, Thermo Fisher Scientific). For first-strand cDNA synthesis, 2 μg of total RNA was reverse-transcribed using a reverse transcriptase enzyme (RiverTraAce, Toyobo, Osaka, Japan) in a 20 μL reaction mixture, according to the manufacturer’s protocol. The resulting cDNA was used as a template for quantitative real-time PCR (qRT-PCR) to analyze the target mRNA expression levels. Real-time PCR was performed using gene-specific primers and Power SyBr Green PCR master mix (Applied Biosystems, Warrington, UK) on a real-time PCR system (Applied Biosystems). The primers for the target genes were designed based on the sequences of rat mRNA. GAPDH mRNA was used as an internal control for normalization, and the relative mRNA expression levels were calculated using the relative quantification method. The data were expressed as fold changes relative to a calibrator sample, where a rat sample of WKY-ND was used as such.

### 4.10. The Western Blot Analysis

Total protein was isolated from the renal cortical areas of the rats (*n* = 3 per group) using ice-cold RIPA buffer (PBS, pH 7.4, 1% Nonidet P-40, 0.5% sodium deoxycholate, 0.1% SDS, 10 mg/mL PMSF, and 1 mg/mL aprotinin). The kidney tissues were homogenized in RIPA buffer at a ratio of 20× (*w*/*v*) using a tissue homogenizer, and the lysates were kept on ice to minimize protein degradation. The homogenates were centrifuged at 12,000× *g* for 15 min at 4 °C, and the supernatants were collected as total protein extracts.

The protein concentration was determined using a bicinchoninic acid (BCA) protein assay kit (Thermo Fisher Scientific) according to the manufacturer’s instructions. Equal amounts of protein (20–60 μg) from each sample were resolved using SDS–polyacrylamide gel electrophoresis (SDS-PAGE) and subsequently transferred onto a polyvinylidene fluoride (PVDF) membrane (Millipore, Billerica, MA, USA) using a wet transfer system. The membrane was blocked for 1 h at room temperature in Blotto A solution (5% non-fat dry milk and 0.5% Tween-20 in TBS) to minimize non-specific binding. Following blocking, the membrane was incubated with a primary antibody specific to the target protein overnight at 4 °C. The membrane was then washed three times with Blotto A, with each wash lasting 5 min, to remove the unbound primary antibody. Next, the membrane was incubated with HRP-conjugated species-specific secondary antibodies (Bio-Rad, Goettingen, Germany) for 1 h at room temperature. After incubation, the membrane was washed three times with Blotto B (0.5% Tween-20 in TBS) and then rinsed in TBS to ensure the removal of excess secondary antibodies. Immunoreactive proteins were detected using an enhanced chemiluminescence (ECL) detection kit (Amersham, England, UK) as per the manufacturer’s protocol. The protein bands were visualized using a chemiluminescence scanner (ImageQuant, Cytiva, Marlborough, MA, USA), and the intensity of the immunoreactive bands was quantified using ImageJ software. To normalize the target protein expression, the membrane was stripped of the target protein antibody using stripping buffer (2% SDS, 62.5 mM Tris-HCl, pH 6.8, and 100 mM β-mercaptoethanol) at 50 °C for 30 min with gentle agitation. The stripped membrane was re-probed with an anti-β-actin antibody, serving as a loading control.

### 4.11. Genetic Analysis of the DNA Fragment Related to Kidney Fibrosis in SHR-SP Rats

A previous study showed that independent of hypertension, a DNA fragment in chromosome 18 (D18Rat73–D18Rat11) from SHR-SP rats is associated with kidney fibrosis [40]. The list of genes within this DNA fragment was retrieved from the Rat Genome Database (RGD, https://rgd.mcw.edu/, accessed on 29 January 2025) (see Supplemental Materials). A Gene Ontology (GO) analysis was performed using the Database for Annotation, Visualization, and Integrated Discovery (DAVID; https://davidbioinformatics.nih.gov/, accessed on 30 January 2025). An enrichment analysis was conducted for the biological process (BP), cellular component (CC), and molecular function (MF) categories to determine the association of specific genes with each GO term (see the Supplementary Materials). The frequency of the GO term assignments for each gene was then quantified.

### 4.12. The Statistical Analysis

All numerical data are presented as the mean ± SD. The statistical analysis was performed using a one-way ANOVA, followed by Scheffé’s post hoc test, or a paired *t*-test. A *p* value of <0.05 was considered statistically significant. All analyses were conducted using IBM SPSS Statistics version 26.0.

## 5. Conclusions

In conclusion, moderate hypertension has minimal effects on kidney fibrosis, whereas an HFD promotes fibrosis through senescence, inflammation, and TGF-β and PDGF-β signaling in normotensive and moderately hypertensive conditions. In severe hypertension, these pathways are already highly activated, leading to extensive fibrosis, with an HFD having little additional impact at early time points. These findings provide valuable insights into the mechanisms of kidney fibrosis and may help develop targeted strategies for managing CKD in the context of hypertension and HFD.

## Data Availability

All of the data from this study has been used to prepare the manuscript.

## Supplementary Materials

The following supporting information can be downloaded at https://www.mdpi.com/article/10.3390/ijms26168031/s1.
