# Supplementary material for: A High-Fat Diet Increases Kidney Fibrosis Through Regulating TGF-β and PDGF-β Signaling Pathways in Normotensive and Hypertensive Rat Models
Source: Int J Mol Sci. 2025 Aug 20;26(16):8031. doi: 10.3390/ijms26168031 (PMC12386213; doi:10.3390/ijms26168031)
Supplement: Supplementary file 1 [file ijms-26-08031-s001.zip › Supplemental figure legend-IJMS.pdf]

Supplemental figure S1

Changes in kidney histology after a high-fat diet feeding at high magnification: To evaluate histological changes in the kidney tubules of WKY, SHR, and SHR-SP rats fed either a control or high-fat diet, hematoxylin and eosin staining was done. Tubular changes were examined under a light microscope at 1000× magnification. The photomicrographs in the upper panel show kidney sections from WKY, SHR, and SHR-SP rats fed a control diet, while those in the lower panel show kidney sections from WKY, SHR, and SHR-SP rats fed a high-fat diet.

Supplemental figure S2

Changes in mesenchymal expansion in the rat kidney after a high-fat diet feeding: For the evaluation mesenchymal expansion, smooth muscle cell α-actin was used as mesenchymal marker. The photomicrographs in the upper panel show kidney sections from WKY, SHR, and SHR-SP rats fed a control diet, while those in the lower panel show kidney sections from WKY, SHR, and SHR-SP rats fed a high-fat diet.

Supplemental figure S3

Changes in mesenchymal and epithelial cell markers in the rat kidneys following high-fat diet feeding: Vimentin and E-cadherin were used as markers for mesenchymal and epithelial cells, respectively. (A) shows the representative immunostaining photomicrographs of vimentin; and representative immunostaining photomicrographs of E-cadherin are shown in (B). In both (A) and (B), the upper panels show kidney sections from WKY, SHR, and SHR-SP rats fed a control diet, while the lower panels show kidney sections from the same rat strains fed a high-fat diet.

Supplemental figure S4

Changes in TGFβR2 protein levels in rat kidneys following high-fat diet feeding: TGFβR2 protein expression in the kidneys of WKY, SHR, and SHR-SP rats fed either a control or high-fat diet was evaluated by Western blotting using a TGFβR2-specific antibody. Following TGFβR2 detection, the membrane was stripped and re-probed with an anti–β-actin antibody. (A) Representative Western blot image showing TGFβR2 and

β-actin. β-actin was used as a loading control. Both TGFβR2 and β-actin were quantified by densitometric analysis, and β-actin-normalized average values of TGFβR2 are shown in (B). Statistical significance is indicated as follows: $^{*}p < 0.05$ compared to control diet-fed rats of the same strain; $^{\#}p < 0.05$ compared to control diet-fed WKY rats. $^{\dagger}p < 0.05$ compared to control diet-fed SHR rats.

Supplemental figure S5

Changes in PDGFRβ protein levels in rat kidneys following high-fat diet feeding: PDGFRβ protein expression in the kidneys of WKY, SHR, and SHR-SP rats fed either a control or high-fat diet was evaluated by Western blotting using a PDGFRβ-specific antibody. Following PDGFRβ detection, the membrane was stripped and re-probed with an anti–β-actin antibody. (A) Representative Western blot image showing PDGFRβ and β-actin. β-actin was used as a loading control. Both PDGFRβ and β-actin were quantified by densitometric analysis, and β-actin-normalized average values of PDGFRβ are shown in (B). Statistical significance is indicated as follows: $^{*}p < 0.05$ compared to control diet-fed rats of the same strain.
