# Supplementary material for: A High-Fat Diet Increases Kidney Fibrosis Through Regulating TGF-β and PDGF-β Signaling Pathways in Normotensive and Hypertensive Rat Models
Source: Int J Mol Sci. 2025 Aug 20;26(16):8031. doi: 10.3390/ijms26168031 (PMC12386213; doi:10.3390/ijms26168031)
Supplement: Supplementary file 1 [file ijms-26-08031-s001.zip › Supplemental figures-IJMS.pptx]

## Slide 1
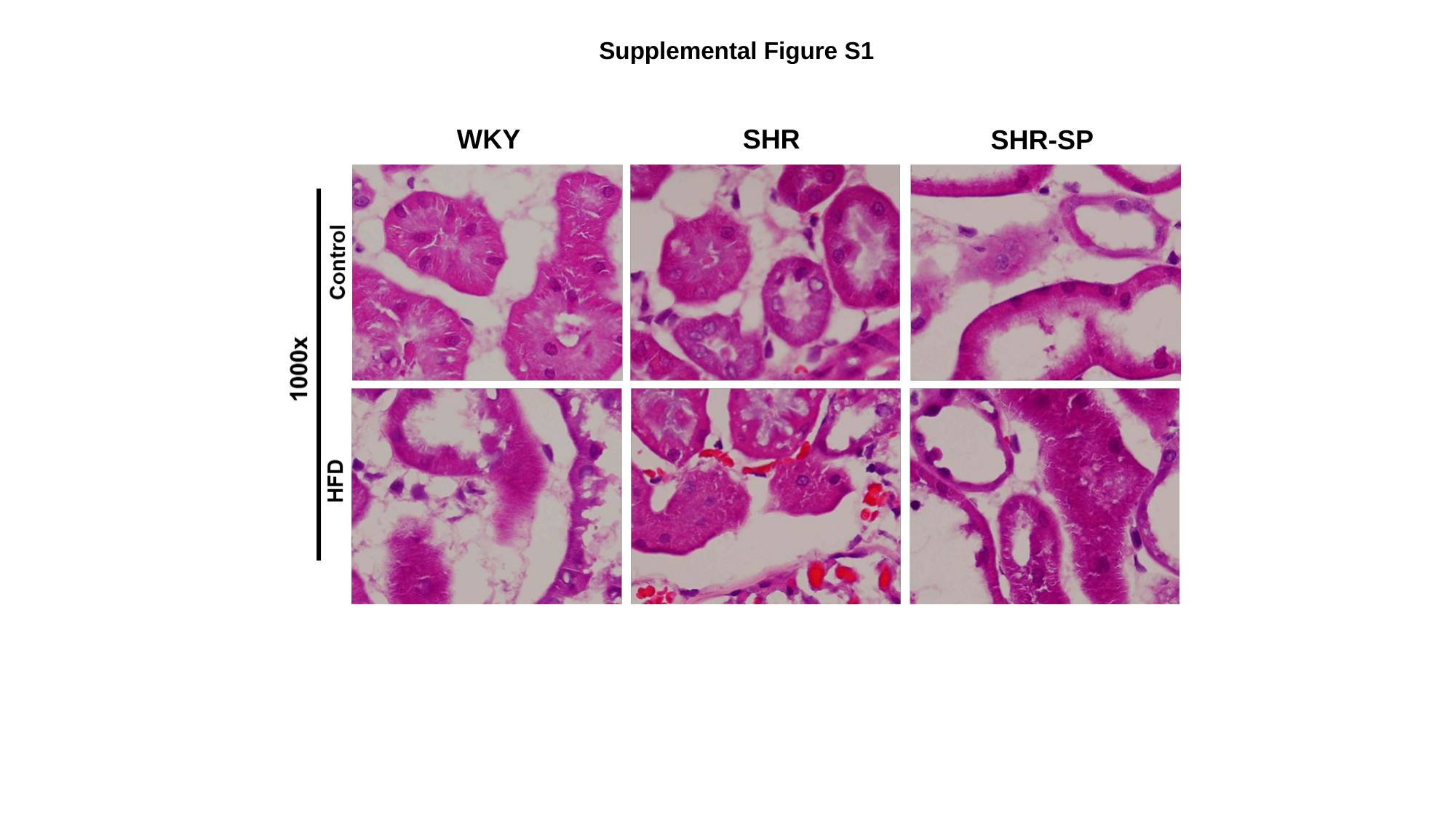

Supplemental Figure S1
SHR
WKY
SHR-SP

## Slide 2
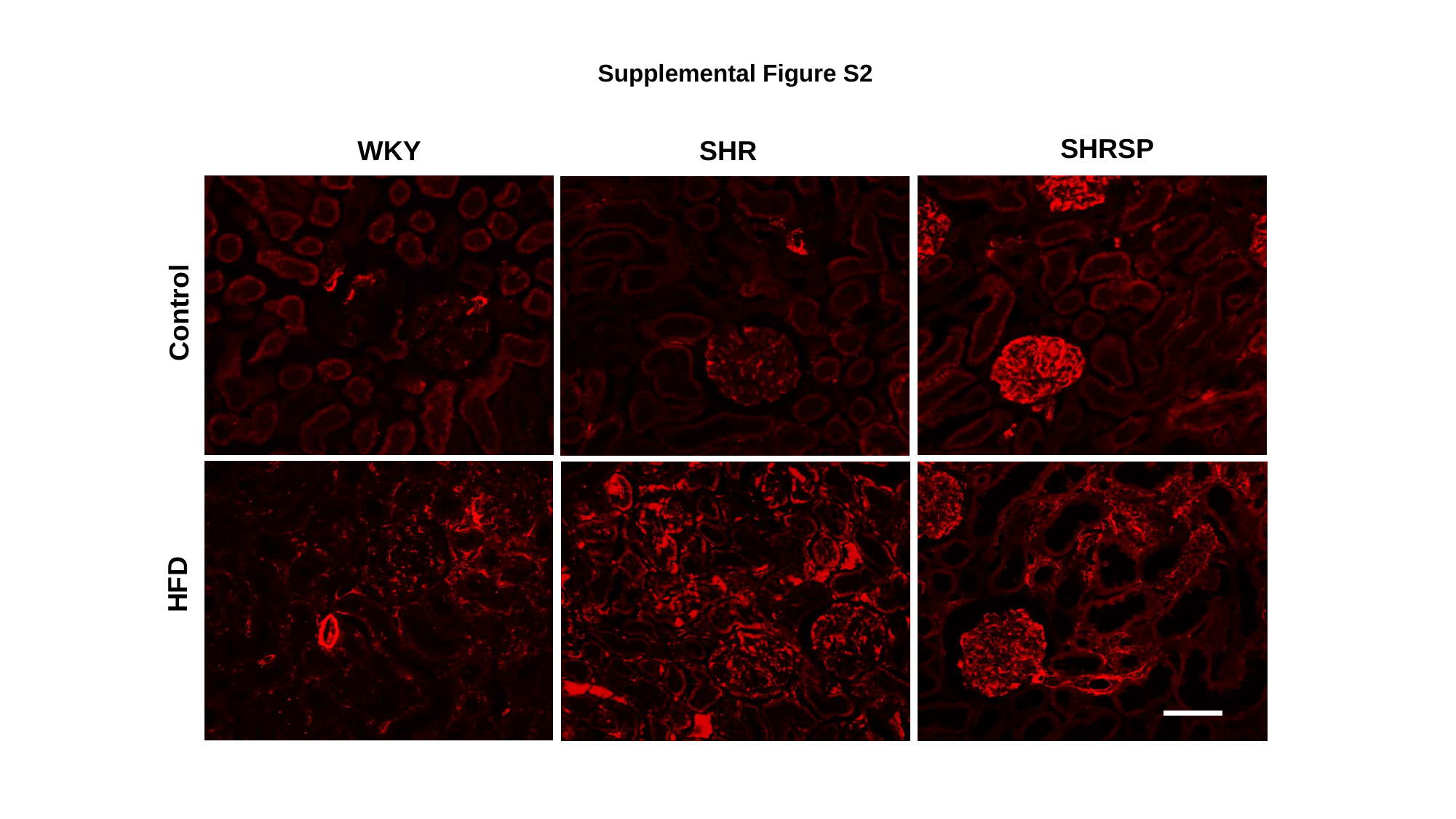

Supplemental Figure S2
SHRSP
SHR
WKY
Control
HFD

## Slide 3
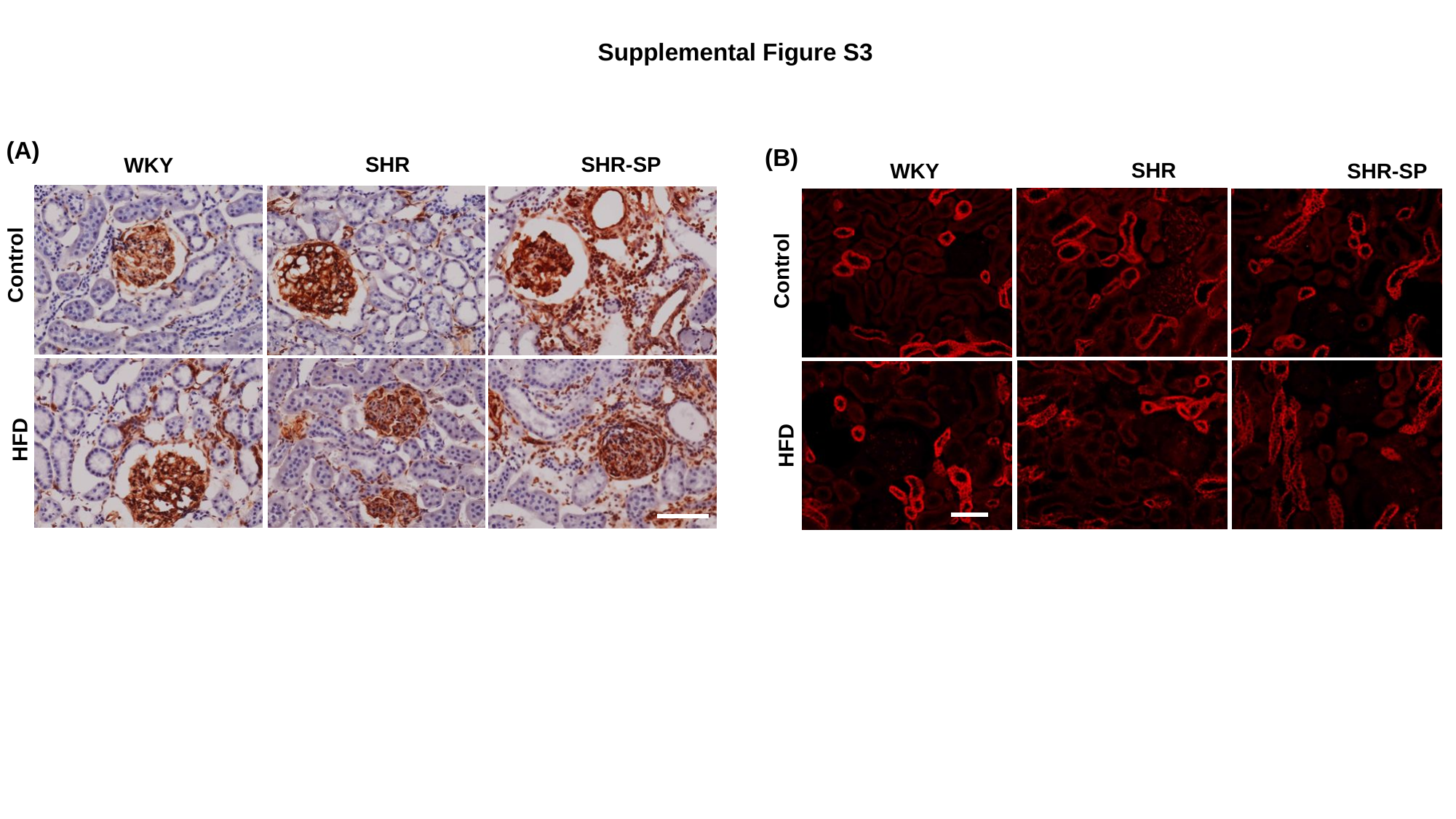

Supplemental Figure S3
(A)
(B)
SHR
SHR-SP
WKY
SHR
SHR-SP
WKY
Control
Control
HFD
HFD

## Slide 4
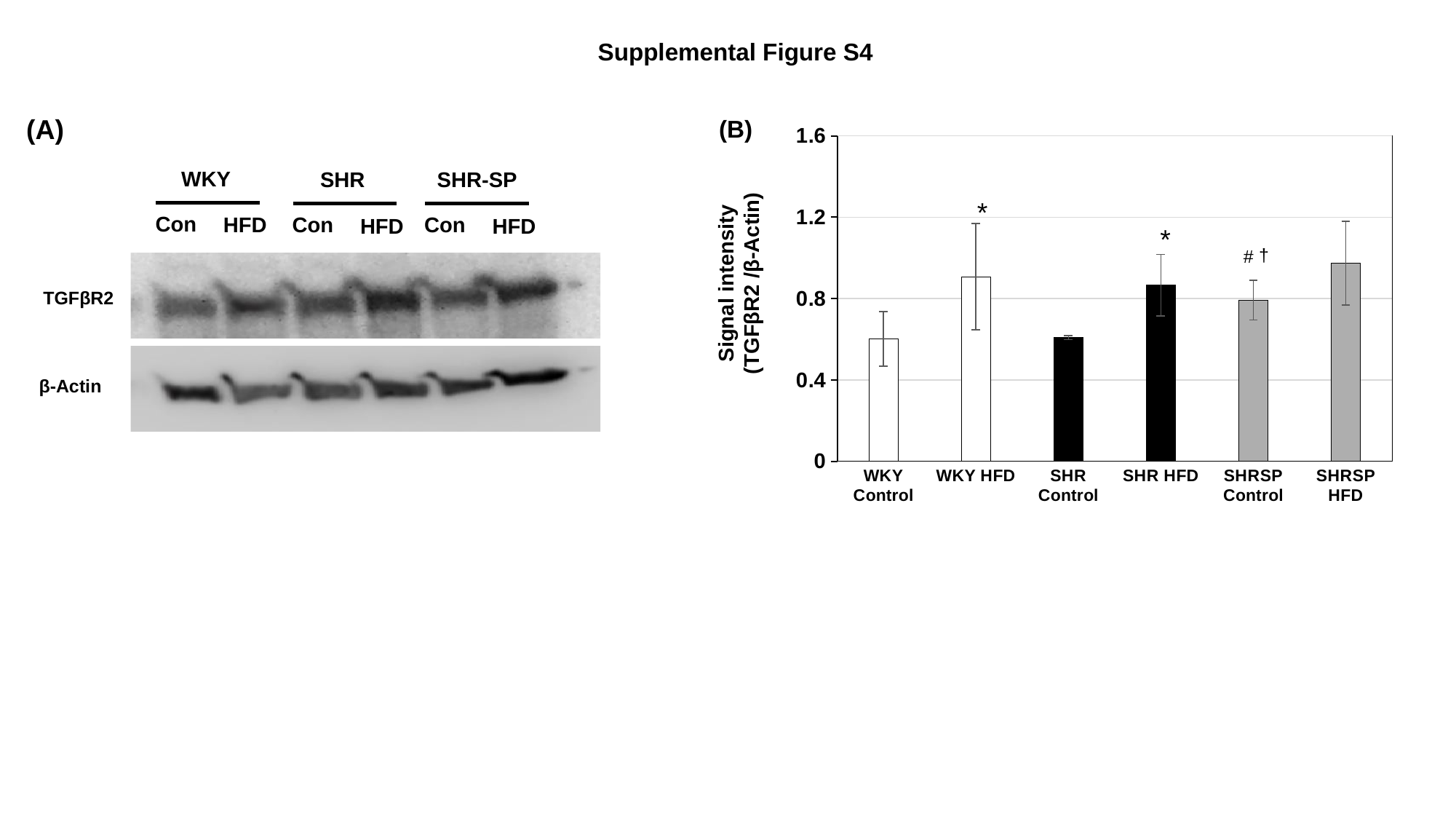

Supplemental Figure S4
(A)
WKY
SHR
SHR-SP
Con
HFD
Con
HFD
Con
HFD
TGFβR2
β-Actin
(B)
### Chart
| Category | |
|---|---|
| WKY Control | 0.6020735851193281 |
| WKY HFD | 0.9076357417085813 |
| SHR Control | 0.6082715076728916 |
| SHR HFD | 0.8654491561359676 |
| SHRSP Control | 0.7927163313041875 |
| SHRSP HFD | 0.9745217547540871 |*
*
†
#
Signal intensity
(TGFβR2 /β-Actin)

## Slide 5
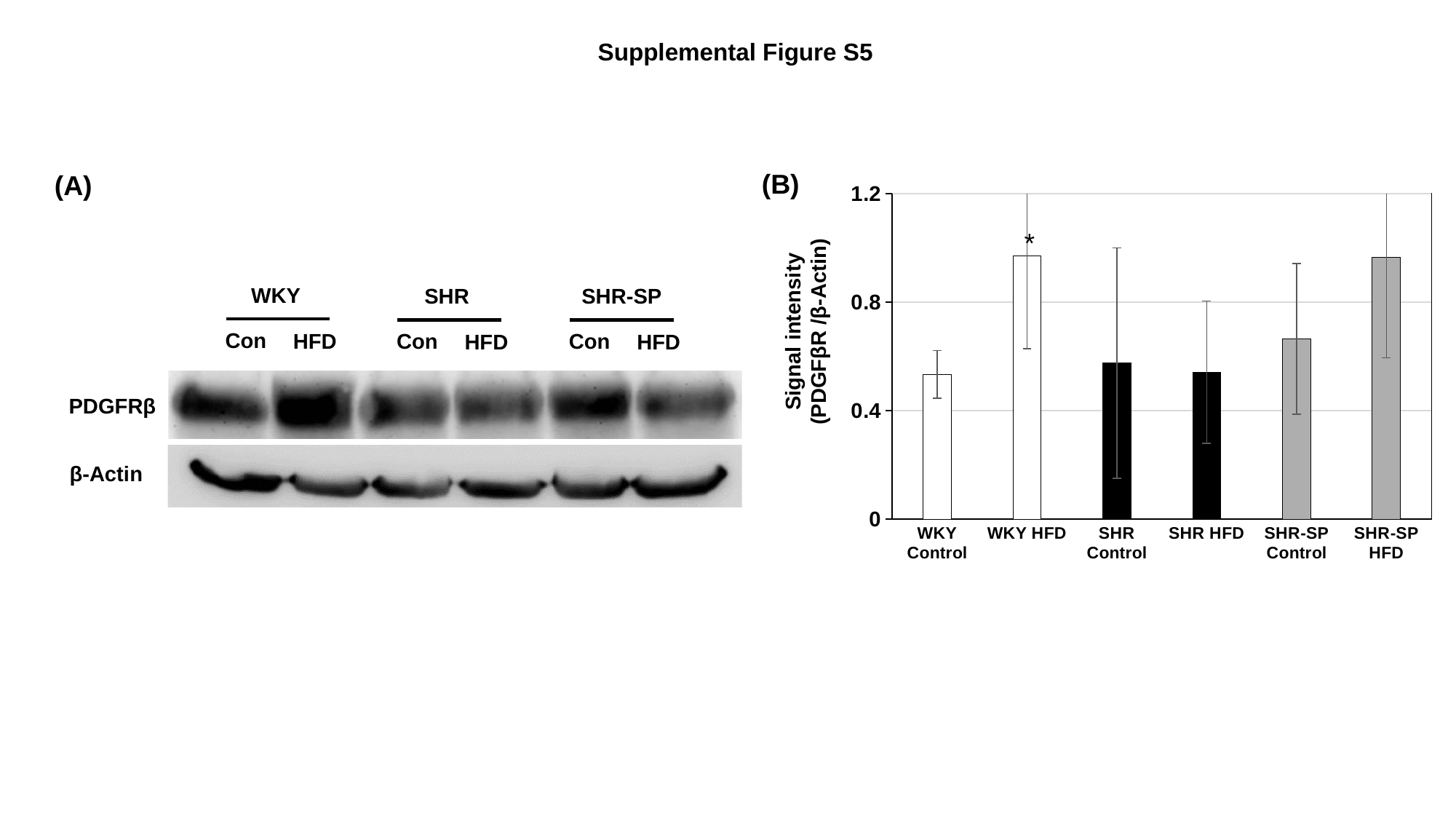

Supplemental Figure S5
(B)
(A)
### Chart
| Category | |
|---|---|
| WKY Control | 0.5331886113534628 |
| WKY HFD | 0.9711226007246766 |
| SHR Control | 0.5751241796877672 |
| SHR HFD | 0.5415552395639076 |
| SHR-SP Control | 0.6643933985270648 |
| SHR-SP HFD | 0.9659937093114715 |*
WKY
SHR
SHR-SP
Signal intensity
(PDGFβR /β-Actin)
Con
HFD
Con
HFD
Con
HFD
PDGFRβ
β-Actin
